# Ordered Co^III^‐MOF@Co^II^‐MOF Heterojunction for Highly Efficient Photocatalytic Syngas Production

**DOI:** 10.1002/smsc.202200085

**Published:** 2023-02-28

**Authors:** Mingxiong Lin, Weishan Jiang, Tingshi Zhang, Bixia Yang, Zanyong Zhuang, Yan Yu

**Affiliations:** ^1^ College of Materials Science and Engineering Fuzhou University New Campus Minhou Fujian 350108 China; ^2^ Key Laboratory of Advanced Materials Technologies Fuzhou University Fuzhou 350108 China

**Keywords:** CO_2_ reduction, mesocrystal, metal–organic framework, ordered heterostructure, syngas production

## Abstract

The design of advanced metal–organic framework (MOF) catalysts for solar‐driven conversion of CO_2_ into syngas (CO/H_2_ mixture) is beneficial. Herein, the design of a joint MOF heterostructure consisting of orderly assembled Co^II^‐ and Co^III^‐based Prussian blue analogs (PBAs) driven by their spontaneous lattice match in the growth process is reported. As‐prepared H/Co^III^‐PBA@Co^II^‐PBA cage is a mesocrystal and exhibits superior photocatalytic syngas production activity (*V*_CO_ up to 50.56 mmol g^−1^ h^−1^, CO/H_2_ = 1:1), which is among the best state‐of‐the‐art heterogeneous photocatalysts in the literature. Theoretical calculations and experimental results confirm that Co^III^‐PBA exerts a stronger affinity for CO_2_ molecules than Co^II^‐PBA, thus serving as the active site. The built‐in electric field in the Co^III^‐PBA@Co^II^‐PBA heterojunction can direct the fast transport of photogenerated electrons from Co^II^‐PBA to the active Co^III^‐PBA. In the present case, the engineering of electronics outweighs morphological engineering to enhance the catalytic properties of Co^III^‐MOF@Co^II^‐MOF for CO_2_‐to‐syngas conversion.

## Graphical abstract

An ordered Co^III^‐MOF@Co^II^‐MOF heterostructure based on a spontaneous‐orientation attachment mechanism is constructed and it is revealed that engineering the electronics of Co^II^/Co^III^ sites in the present case outweighs morphological engineering to enhance the catalytic properties of Co^III^‐MOF@Co^II^‐MOF for CO_2_‐to‐syngas conversion.

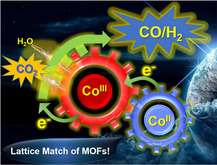

## 1 Introduction

Syngas, composed of CO and H_2,_ is a key feedstock for preparing value‐added chemicals essential to modern society.^[^ ^1^ ^]^ For example, syngas is the starting material for the thermochemical synthesis of methanol and formic acid and is used in the Fischer–Tropsch process to produce liquid hydrocarbons.^[^ ^2^ ^]^ Current technologies use coal and natural gas to prepare syngas under harsh conditions (e.g., 700–1000 °C and 3–25 atm),^[^ ^3^ ^]^ and obtaining syngas from atmospheric CO_2_ using a green energy source can decrease the carbon footprint of the established chemical industry,^[^ ^4^ ^]^ hence developing advanced catalysts that can harvest solar energy to reduce inert CO_2_ to CO and H_2_O to H_2_; thus, the generation of syngas is highly appealing.

Many metal–organic frameworks (MOFs) are excellent photocatalysts.^[^ ^5^ ^]^ In particular, there have been significant advances in exploiting cobalt‐based MOFs (Co‐MOFs) as novel catalysts for the CO_2_ reduction reaction (CRR) and hydrogen evolution reaction (HER)[^5^, ^6^] because the loosely bonded d electrons at the active Co sites can readily engage in photocatalytic processes.^[^ ^7^ ^]^ Co‐MOFs sometimes preferentially engage in the CRR (e.g., MOF‐525‐Co,^[^ ^8^ ^]^ Co‐ZIF‐9,^[^ ^9^ ^]^ and [Co_3_(HL)_2_·4DMF·4H_2_O]^[^ ^10^ ^]^) or the HER (e.g., CoP/Co‐MOF,^[^ ^11^ ^]^ Co‐Cl_4_‐MOF,^[^ ^12^ ^]^ and Co/Cu‐MOF^[^ ^13^ ^]^), whereas in other cases (e.g., (Co/Ru)_2.4_‐UiO‐67(bpydc)^[^ ^14^ ^]^ and ZIF‐67^[^ ^15^ ^]^), the cogeneration of CO and H_2_ has been demonstrated. An appealing pathway is to tailor the oxidation state of the active Co sites finely to customize the performance of Co‐MOF photocatalysts.^[^ ^16^ ^]^ It has been envisioned that a relatively high oxidation state of Co improves CO_2_ adsorption on the catalyst to achieve efficient CRR,^[^ ^17^ ^]^ whereas a low oxidation state of Co may promote HER.^[^ ^18^ ^]^ In addition, charge recombination can be suppressed by having an appropriate Co oxidation state,^[^ ^19^ ^]^ and combining MOF components with different Co oxidation states is expected to enhance the charge transport kinetics.^[^ ^20^ ^]^ Heterostructures in which two Co‐MOFs of different Co oxidation states are joined together may be suitable for cogenerating CO and H_2_.

Constant attention has been given to the interface engineering of Co^II^/Co^III^ in Co‐MOFs, which is also crucial for unraveling the underlying form‐to‐function relationship and predicting the catalytic properties of the MOF heterostructure. An ideal configuration should have an ordered assembly of Co^II^‐MOF/Co^III^‐MOF in intimate contact, which can optimize the Co^II^/Co^III^‐related electrochemically active sites and the charge transfer pathway/kinetics of the MOFs heterostructure. While some progress has been made in developing an MOF hybrid and engineering the core–shell structure,^[^ ^21^ ^]^ preparing orderly assembled MOFs with different Co oxidation states on each MOF building block. In brief, the lack of control over the metal sites in MOFs limits the fine‐tuning of heterostructures for advanced catalysis. In addition, studies have reported the morphological engineering of MOFs, for example, building cage‐like structures, whereas they mostly suggested that the large cavity of the cage may favor the catalytic performance of MOFs.[^20^, ^22^] In contrast, the electronic engineering of MOFs has not been adequately explored.

In this work, we prepared ordered Co^III^‐MOF@Co^II^‐MOF heterojunctions with intimate contact, in which the two Co‐MOFs (with a Prussian blue analog [PBA] as an example) have distinct valence Co states but spontaneously assembled through oriented attachment, forming a Co^III^‐PBA/Co^II^‐PBA mesocrystal. Specifically, Co^III^‐PBA forms a cage to encapsulate Co^II^‐PBA, forming either a cubic block or a smaller cage. The mechanism behind the excellent performance of Co^III^‐PBA@Co^II^‐PBA in syngas generation (*V*_CO_ = 50 mmol g^−1^ h^−1^, CO/H_2_ = 1:1) was explored by experimental characterization along with density functional theory (DFT) calculations.

## 2 Results and Discussion

### 2.1 Establishment of Different Co^III^‐PBA@Co^II^‐PBA Structures

**Figure 1:**
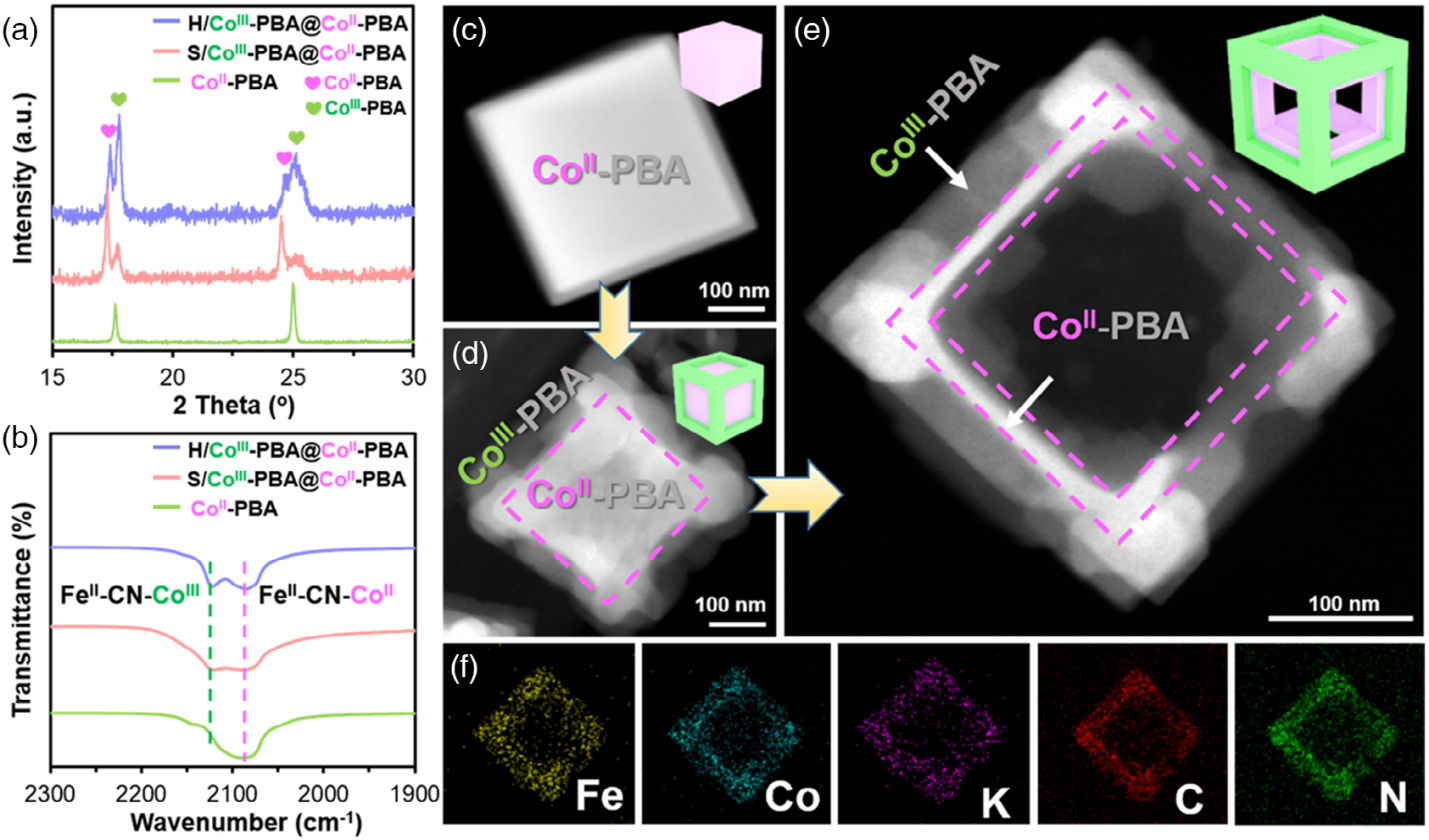
a) XRD patterns and b) FT‐IR spectra of Co^II^‐PBA, S/Co^III^‐PBA@Co^II^‐PBA, and H/Co^III^‐PBA@Co^II^‐PBA; c–e) HAADF‐STEM images of Co^II^‐PBA (c), S/Co^III^‐PBA@Co^II^‐PBA (d), and H/Co^III^‐PBA@Co^II^‐PBA (e). f) Elemental mappings of H/Co^III^‐PBA@Co^II^‐PBA.

Different Co‐PBA structures were obtained using the same procedure by varying the stoichiometry of the starting materials, that is, Co(CH_3_CO_2_)_2_ and K_3_Fe(CN)_6_. When the content of Co(CH_3_CO_2_)_2_ was low concerning K_3_Fe(CN)_6_, a single‐component PBA that can be identified as K_2_Co[Fe(CN)_6_] (JCPDS No.75‐0038) from X‐ray diffraction (XRD) was obtained (**Figure** **1**a and Figure S1a, Supporting Information), hereafter referred to as Co^II^‐PBA. The Fourier transform infrared (FT‐IR) spectrum (Figure 1b and Figure S1b, Supporting Information) of Co^II^‐PBA displays a CN vibration peak at ≈2083 cm^−1,^ which can be ascribed to Co^II^–NC–Fe^II^.^[^ ^23^ ^]^ Increasing the Co^2+^ content in the starting materials gives rise to a new set of XRD peaks, whose profile is analogous to that of Co^II^‐PBA but whose peak positions shift to higher angles, indicating a contraction of cell parameters (Figure 1a and S1a, Supporting Information). The FT‐IR spectra revealed a new CN vibration peak at ≈2120 cm^−1^ which can be related to Co^III^–NC–Fe^II^ (Figure 1b and S1b, Supporting Information). The results of elemental analysis and energy‐dispersive spectrometry (EDS, Figure S2, Supporting Information) showed a reduced K content when compared to pristine Co^II^‐PBA (K_2_Co[Fe(CN)_6_]). Therefore, the new phase can be deemed KCo[Fe(CN)_6_] (hereafter referred to as Co^III^‐PBA), and the obtained product is a heterostructure comprising Co^II^‐PBA and Co^III^‐PBA.

In the scanning electron microscopy (SEM) (Figure S3a, Supporting Information) and transmission electron microscopy (TEM) images (Figure 1c), pure Co^II^‐PBA appeared as solid cubes with a uniform particle size of approximately ≈400 nm (Figure S3b,c, Supporting Information). Each Co^II^‐PBA cube was a single crystal with a spot‐like selected‐area electron diffraction (SAED) pattern (Figure S3d, Supporting Information), and the K, Fe, and Co elements were distributed homogeneously in the C and N matrices (Figure S4, Supporting Information). The morphology of the Co^III^‐PBA@Co^II^‐PBA heterostructure depended on the Co^2+^ dosage in the starting materials during material synthesis. The SEM and TEM images (Figure 1d and S5, Supporting Information) demonstrate that when the Co/Fe ratio of the starting materials was 1.50, additional Co^III^‐PBA was decorated on the edge of the previously described solid Co^II^‐PBA cube, yielding a product hereafter referred to as S/Co^III^‐PBA@Co^II^‐PBA. The Co^II^‐PBA cube was enclosed in a Co^III^‐PBA cage, whose columns were ≈80 nm thick. When the Co/Fe ratio of the starting materials was further increased to 2.20, the obtained heterostructure no longer had an enclosed Co^II^‐PBA cube but consisted of a Co^II^‐PBA cage inside a larger Co^III^‐PBA cage (Figure 1e and S6, Supporting Information). However, the particle size of the heterostructure (hereafter referred to as H/Co^III^‐PBA@Co^II^‐PBA) was unchanged, as each Co^II^‐PBA cage was still ≈400 nm in length. The columns of the Co^II^‐PBA and Co^III^‐PBA cages were ≈30 and ≈80 nm thick, respectively (Figure S7, Supporting Information). The outer Co^III^‐PBA cage was in intimate contact with the inner Co^II^‐PBA cage, as can be seen from the high‐resolution TEM (HRTEM) image (Figure S8, Supporting Information). Elemental mapping (Figure 1f and S9, Supporting Information) showed a uniform distribution of K, Fe, Co, C, and N throughout the heterostructure for both S/Co^III^‐PBA@Co^II^‐PBA and H/Co^III^‐PBA@Co^II^‐PBA.

**Figure 2:**
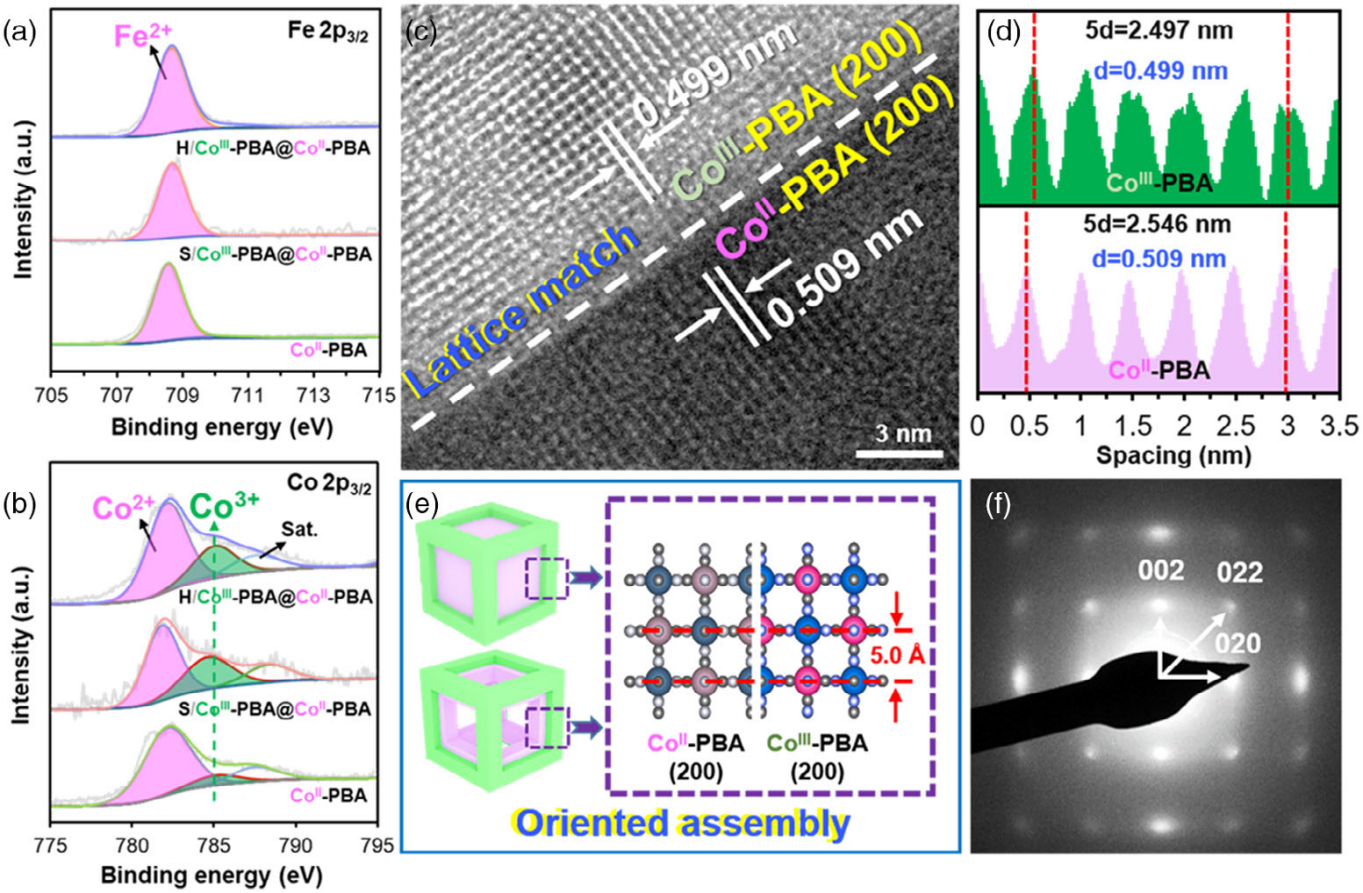
a,b) The XPS spectra of Fe 2p_3/2_ (a) and Co 2p_3/2_ (b) of Co^II^‐PBA, S/Co^III^‐PBA@Co^II^‐PBA, and H/Co^III^‐PBA@Co^II^‐PBA. c) HRTEM image and d) corresponding lattice fringe spacing of H/Co^III^‐PBA@Co^II^‐PBA. e) Lattice matching of Co^III^‐PBA and Co^II^‐PBA. f) SAED pattern of H/Co^III^‐PBA@Co^II^‐PBA.

The Co/Fe ratio can be determined from EDS to be 1.39 for Co^II^‐PBA, 1.22 for S/Co^III^‐PBA@Co^II^‐PBA, and 1.59 for H/Co^III^‐PBA@Co^II^‐PBA (Figure S10, Supporting Information). The X‐ray photoelectron spectroscopy (XPS) images of Co^II^‐PBA, H/Co^III^‐PBA@Co^II^‐PBA, and S/Co^III^‐PBA@Co^II^‐PBA show similar Fe 2p_3/2_ spectra, with peaks at 708.7 eV ascribed to the presence of Fe^2+^.^[^ ^24^ ^]^ In the Co 2p_3/2_ spectra, Co^2+^ appears at 782.1 eV, and Co^3+^ appears at 785.1 eV (**Figure** **2**a). The XPS peak of Co^3+^ intensified significantly upon introducing Co^III^‐PBA (Figure 2b).

Therefore, the formation of Co^III^‐PBA@Co^II^‐PBA can be rationalized as follows: as the redox potential of Co^2+^/Co^3+^ (−1.83 eV vs normal hydrogen electrode (NHE)) is lower than that of [Fe^3+^(CN)_6_]^3−^/[Fe^2+^(CN)_6_]^4−^ (0.36 eV vs NHE) (Figure S11, Supporting Information), the oxidation of Co^2+^ by [Fe^3+^(CN)_6_]^3−^ will give Co^3+^ and [Fe^2+^(CN)_6_]^4−^. The reaction between Co^2+^ and [Fe^2+^(CN)_6_]^4−^ afforded Co^II^‐PBA (Co^II^–NC–Fe^II^). When the starting materials had a low Co/Fe ratio of 0.20, the relatively small amount of Fe^2+^ more readily joined Co^II^ to give Co^II^–NC–Fe^II^ as the precipitate, probably because of the relatively low solubility of Co^II^‐PBA. In the PBA structure, electron redistribution can occur between the two metal sites, with cyanide (−CN) groups as the bridge.^[^ ^25^ ^]^ When the Co/Fe ratio in the starting materials increased to 1.50, Co^III^‐PBA (Co^III^–NC–Fe^II^) was formed when [Fe^2+^(CN)_6_]^4−^ reacted with Co^3+^. The precipitation of Co^III^‐PBA (Co^III^–NC–Fe^II^) becomes feasible, followed by the formation of Co^II^–NC–Fe^II^, spontaneously creating a heterostructure. This reveals that MOFs can regulate their growth behavior to form intriguing and complex cages or solid heterostructures of assembled architectures.

### 2.2 Oriented Assembly of Co^III^‐PBA on Co^II^‐PBA

Co^II^‐PBA and Co^III^‐PBA have well‐resolved lattice fringes in the HRTEM image (Figure 2c,d, and S12, Supporting Information), *d*_(200)_ = 0.499 and 0.509 nm for Co^III^‐PBA and Co^II^‐PBA, respectively. The observed *d* spacings indexed to the diffraction of the (200) facets of PBA agree well with the results calculated from XRD, that is, *d*_(200)_ = 0.498 and 0.509 nm for Co^III^‐PBA and Co^II^‐PBA, respectively. There is an evident oriented attachment of two distinct building blocks (difference in Co valences), as 1) the (200) facets of Co^III^‐PBA are parallel to the (200) facets of Co^II^‐PBA, and 2) on each edge of the cage‐in‐cage architecture, the outer Co^III^‐PBA always shares the same orientation as the inner Co^II^‐PBA (Figure 2e and S13, Supporting Information). As a result, both S/Co^III^‐PBA@Co^II^‐PBA and H/Co^III^‐PBA@Co^II^‐PBA can be identified as mesocrystals, which can diffract electrons of the Co^II^‐PBA single crystal. In addition, all the samples displayed the typical <002>, <022>, and <020> zone axes of cubic PBA (Figure 2f and S14, Supporting Information).

**Figure 3:**
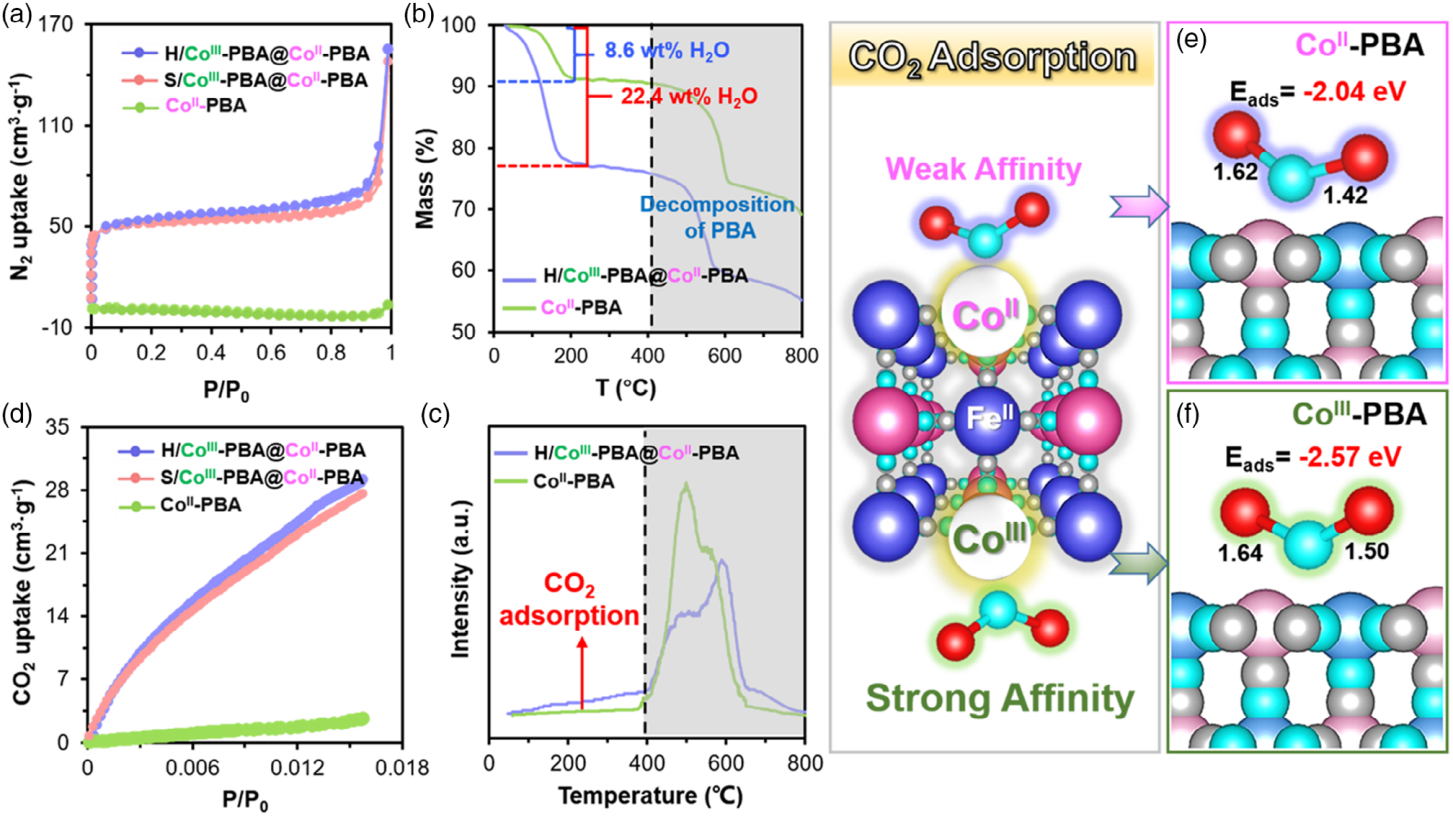
a) BET surface area analysis of Co^II^‐PBA, S/Co^III^‐PBA@Co^II^‐PBA, and H/Co^III^‐PBA@Co^II^‐PBA. b) TG analysis and c) CO_2_‐TPD measurement of Co^II^‐PBA and H/Co^III^‐PBA@Co^II^‐PBA. d) CO_2_‐uptake capability of Co^II^‐PBA, S/Co^III^‐PBA@Co^II^‐PBA, and H/Co^III^‐PBA@Co^II^‐PBA. e,f) Binding and activation of CO_2_ on the surface of the modeled Co^II^‐PBA (Co^II^–N ≡ C–Fe^II^) (e) and Co^III^‐PBA (Co^III^–N ≡ C–Fe^II^) (f).

The Brunauer–Emmett–Teller (BET)‐specific surface area of Co^II^‐PBA (1.4 cm^2^ g^−1^) is much lower than that of S/Co^III^‐PBA@Co^II^‐PBA (156.6 cm^2^ g^−1^) and H/Co^III^‐PBA@Co^II^‐PBA (162.4 cm^2^ g^−1^) (**Figure** **3**a and Table S1, Supporting Information). There are abundant microspores (≈0.7–1.3 nm in size) in both S/Co^III^‐PBA@Co^II^‐PBA and H/Co^III^‐PBA@Co^II^‐PBA (Figure S15, Supporting Information). The cavity of the hollow H/Co^III^‐PBA@Co^II^‐PBA cage was at the submicrometer scale.

Thermogravimetric (TG) analysis verified that Co^II^‐PBA accommodated much less H_2_O than H/Co^III^‐PBA@Co^II^‐PBA (Figure 3b). Specifically, the dehydration of adsorbed or crystallized water at <200 °C amounts to 8.6 and 22.4 wt% weight loss for Co^II^‐PBA and H/Co^III^‐PBA@Co^II^‐PBA, respectively. After the dehydration process, Co^II^‐PBA and H/Co^III^‐PBA@Co^II^‐PBA had the same decomposition temperature profiles that matched the literature values for the PBA structures.^[^ ^26^ ^]^ The CO_2_ adsorption capacity was measured using CO_2_ temperature‐programmed desorption (CO_2_‐TPD), and the CO_2_‐TPD peak at <400 °C was taken as the release of adsorbed CO_2_ because the PBA structure collapsed at >400 °C. Figure 3c shows that Co^II^‐PBA has a much weaker adsorption capability for CO_2_ than H/Co^III^‐PBA@Co^II^‐PBA. Figure 3d and Table S1, Supporting Information, show that the CO_2_ adsorption capacity of the PBA is positively correlated with the Co^III^–NC–Fe^II^ (Co^III^‐PBA), ranking in the order of H/Co^III^‐PBA@Co^II^‐PBA (29.1 cm^3^ g^−1^) =S/Co^III^‐PBA@ Co^II^‐PBA (27.6 cm^3^ g^−1^) > Co^II^‐PBA (2.7 cm^3^ g^−1^).

The binding and activation of CO_2_ on the surfaces of Co^II^‐PBA (Co^II^–NC–Fe^II^) and Co^III^‐PBA (Co^III^–NC–Fe^II^) were modeled by DFT calculations. While CO_2_ can be chemically bound to the surfaces of both Co^II^‐PBA and Co^III^‐PBA, the CO_2_ adsorption energy (Δ*E*_ad‐CO2_) is harmful to both Co^II^‐PBA and Co^III^‐PBA; Co^III^‐PBA seems to bind CO_2_ more strongly than Co^II^‐PBA. Figure 3e shows that the bond lengths of CO_2_ are 1.42 and 1.62 Å on Co^II^‐PBA but are elongated to 1.50 and 1.64 Å on Co^III^‐PBA as a result of stronger chemical interaction. Furthermore, Figure 3f shows that Δ*E*_ad‐CO2_ is −2.57 eV for Co^III^‐PBA (Co^III^–NC–Fe^II^) but −2.04 eV for Co^II^‐PBA (Co^II^–NC–Fe^II^). Hence, the DFT results are consistent with the experimental findings and further demonstrate that Co^III^‐PBA (Co^III^–NC–Fe^II^) enhances the adsorption and activation of CO_2_.

### 2.3 Syngas Production

The performance of photocatalytic syngas production was then compared among Co^II^‐PBA, S/Co^III^‐PBA@Co^II^‐PBA, and H/Co^III^‐PBA@Co^II^‐PBA. To this end, the bandgaps of Co^II^ PBA, S/Co^III^ PBA@Co^II^ PBA, and H/Co^III^ PBA@Co^II^ PBA (Figure S16 and Table S2, Supporting Information) were first investigated, estimated to be 2.40, 2.00, and 2.30 eV, respectively, according to the Tauc plot. The position of conduction band minimum (*E*_CBM_) of Co^II^ PBA, S/Co^III^ PBA@Co^II^ PBA, and H/Co^III^ PBA@Co^II^ PBA is also estimated to be −0.87, −0.62, and −0.77 eV, respectively, based on the Mott–Schottky plots (Figure S17, Supporting Information). With the bandgaps’ values, the valence band minimum (*E*_VBM_) positions of Co^II^ PBA, S/Co^III^ PBA@Co^II^ PBA, and H/Co^III^ PBA@Co^II^ PBA were determined to be 1.53, 1.38, and 1.53 eV, respectively. Note that the conduction bands of the samples are thermodynamically favorable for the reduction of CO_2_ to CO (−0.51 eV) and H_2_O to H_2_ (−0.41 eV) (Figure S18, Supporting Information)^[^ ^27^ ^]^ and the photogenerated electrons in the lowest unoccupied molecular orbital (LUMO) of Ru can be sent to the conduction band of PBA for the reduction reaction (Figure S19, Supporting Information).

**Figure 4:**
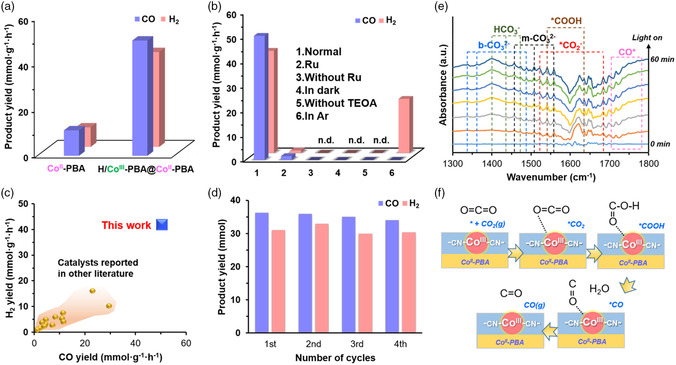
a) Photocatalytic syngas generation over Co^II^‐PBA and H/Co^III^‐PBA@Co^II^‐PBA. b) Gas generation rates under different reaction conditions with H/Co^III^‐PBA@Co^II^‐PBA as the catalyst. c) Performance of H/Co^III^‐PBA@Co^II^‐PBA and other reported catalysts in syngas generation. d) Recycling test of H/Co^III^‐PBA@Co^II^‐PBA. e) In situ FT‐IR spectra for CO_2_ and H_2_O reaction over the H/Co^III^‐PBA@Co^II^‐PBA under light illumination. f) Schematic illustration of the CRR process on the H/Co^III^‐PBA@Co^II^‐PBA.

The photocatalytic reactions were then performed under normal photocatalytic conditions. Here, [Ru(bpy)_3_]Cl_2_·6H_2_O (bpy = 2,2′‐bipyridine) is used as the photosensitizer and triethanolamine (TEOA) as the electron donor. **Figure** **4**a shows that the gas yields were low when Co^II^‐PBA was used (*V*_CO_ = 11.18 mmol g^−1^ h^−1^, *V*_H2_ = 8.59 mmol g^−1^ h^−1^) but improved dramatically for H/Co^III^‐PBA@Co^II^‐PBA (*V*_CO_ = 50.56 mmol g^−1^ h^−1^, *V*_H2_ = 41.63 mmol g^−1^ h^−1^). This enhanced performance presumably arises from Co^III^–NC–Fe^II^ (Co^III^‐PBA), which provides more reactive sites to adsorb CO_2_ and accommodate H_2_O. Because H/Co^III^‐PBA@Co^II^‐PBA and S/Co^III^‐PBA@Co^II^‐PBA cages have identical chemical components with very similar phases and differ only in morphology, they serve as ideal pairs for examining the role of the cage and the active metal sites in the catalytic reaction. It does not seem to be critical to catalyst performance, as S/Co^III^‐PBA@Co^II^‐PBA gives even higher gas yields of *V*_CO_ = 51.2 mmol g^−1^ h^−1^ and *V*_H2_ = 45.8 mmol g^−1^ h^−1^ (Figure S20a, Supporting Information). For both heterostructures, the presence of Co^III^‐PBA significantly improved charge transfer and allowed the redox reaction to proceed efficiently.^[^ ^28^ ^]^

The ^1^H‐NMR measurement ruled out the formation of liquid products, such as CH_3_OH, HCOOH, and HCHO (Figure S20b, Supporting Information), and the isotopic ^13^C‐labelled experiment confirms that ^13^CO was obtained from the reduction of ^13^CO_2_ (Figure S20c, Supporting Information). Figure 4b shows that no gaseous products were formed without light, TEOA, or [Ru(bpy)_3_]Cl_2_·6H_2_O. The gas yield was also negligible without the input of the PBA catalyst, and only H_2_ was obtained when the reaction was conducted under an Ar atmosphere. Notably, the tendency of CO generation matches well with the absorption spectrum of the Ru photosensitizer (Figure S20d, Supporting Information). Hence, the solar‐driven CRR and HER over PBA were driven by the excitation of [Ru(bpy)_3_]Cl_2_·6H_2_O, whose electrons were sent to the active site on PBA for subsequent reduction reactions.^[^ ^29^ ^]^

Photocatalysis using H/Co^III^‐PBA@Co^II^‐PBA generated syngas with a CO/H_2_ ratio of approximately 1:1 (Figure S21, Supporting Information), which can be used for hydroformylation, one of the most common industrial reactions for the production of important chemical commodities.^[^ ^30^ ^]^ When assessed based on the combined gas yield (mmol g^−1^ h^−1^), H/Co^III^‐PBA@Co^II^‐PBA outperforms many state‐of‐the‐art heterogeneous photocatalysts reported in the literature (Figure 4c nd Table S2, Supporting Information), such as Fe–SAs/N–C (*V*_CO_ = 4.50, *V*_H2_ = 4.95),^[^ ^31^ ^]^Co_3_O_4_‐NS (*V*_CO_ = 23.00, *V*_H2_ = 16.12),^[^ ^32^ ^]^ Co‐ZIF‐9 (*V*_CO_ = 8.36, *V*_H2_ = 5.98),^[^ ^9^ ^]^ etc. H/Co^III^‐PBA@Co^II^‐PBA maintained good catalytic activity over four repeated cycles (Figure 4d), and the hollow architecture remained intact in the retrieved catalysts, as confirmed by XRD, FT‐IR, XPS, and SEM (Figure S22, Supporting Information). Thus, the H/Co^III^‐PBA@Co^II^‐PBA cage is a robust catalyst for syngas production.

The catalytically active sites in the heterostructures were further investigated. To this end, control samples, including FeFe PB (without Co) and CoCo PBA (without Fe), were prepared. As shown in Figure S23, Supporting Information, the FeFe PB composed of the Fe element only gave negligible CO production (*V*_CO_ = 2.51 mmol g^−1^ h^−1^), while the Co‐bearing CoCo PBA and FeCo PBA (i.e., H/Co^III^‐PBA@Co^II^‐PBA) gave considerably high CO production efficiency. This indicates that Co sites act as CRR active sites in the heterostructure, while the synergy of Fe and Co in the PBA structure promotes CRR performance. In situ FT‐IR spectroscopy characterization further confirmed the adsorption and activation of CO_2_ on the catalysts (Figure 4e). The result under the light irradiation is as follows: the *CO_2_^−^ species at 1522 and 1685 cm^−1^, monodentate carbonate groups (m‐CO_3_^2−^) at 1459, 1509, and 1560 cm^−1^, bidentate carbonate (b‐CO_3_^2−^) at 1336, 1359, and 1490 cm^−1^, and carbonate group (HCO_3_^2−^) at 1401, 1432, and 1469 cm^−1^ were detected. It indicates the adsorption and activation of CO_2_ on the catalysts to generate the critical intermediates of CO_2_ reduction.^[^ ^33^ ^]^ Furthermore, the IR peaks at 1637 and 1538 cm^−1^ intensified, demonstrating the generation of COOH*, a key intermediate in reducing CO_2_ to CO.^[^ ^34^ ^]^ In addition, the bridged CO* absorption peak at 1700–1800 cm^−1^ was also detected, suggesting CO product generation. Hence, a probable reduction pathway involving “CO_2_ → *CO_2_ → *COOH → *CO → CO” is proposed for the presenting system (Figure 4f).^[^ ^35^ ^]^

### 2.4 Directed Transport of Photogenerated Electrons

**Figure 5:**
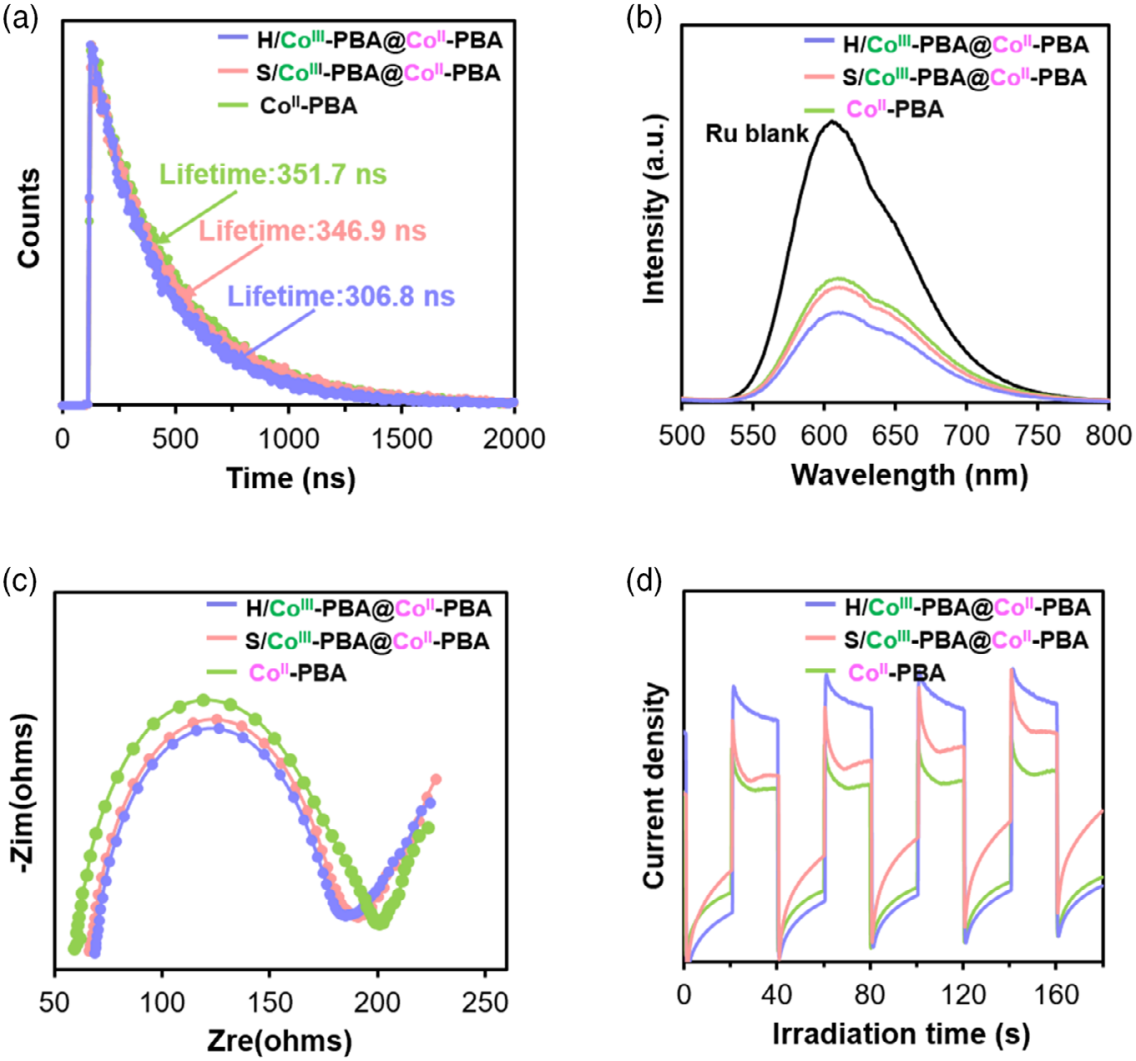
a) TRPL spectra, b) PL spectra, c) EIS measurement, and d) photocurrent of Co^II^‐PBA, S/Co^III^‐PBA@Co^II^‐PBA, and H/Co^III^‐PBA@Co^II^‐PBA.

The enhanced charge transfer in the heterostructures can be observed from the steady‐state photoluminescence (PL) spectra, time‐resolved PL (TRPL) spectra, and photocurrent and electrochemical impedance spectroscopy (EIS) measurements.^[^ ^36^ ^]^ TRPL spectra in **Figure** **5**a confirm the enhanced charge transfer efficiency in the heterostructures^[^ ^37^ ^]^ because H/Co^III^‐PBA@Co^II^‐PBA/Ru (306.8 ns) and S/Co^III^‐PBA@Co^II^‐PBA/Ru (346.9 ns) displayed shorter average lifetimes than Co^II^‐PBA/Ru (351.7 ns). Furthermore, the recombination of light‐excited charge carriers was examined using PL spectroscopy. As shown in Figure 5b, about the photosensitizer [Ru(bpy)_3_]^2+^ with a characteristic emission peak at approximately 607 nm,^[^ ^38^ ^]^ the heterostructures can promote charge transfer, leading to a decrease in the PL intensity in the presence of heterostructures, ranking in the order of H/Co^III^‐PBA@Co^II^‐PBA/Ru < S/Co^III^‐PBA@Co^II^‐PBA/Ru < Co^II^‐PBA/Ru < Ru. The trends of the EIS spectra (Figure 5c) and photocurrent curves (Figure 5d) show that H/Co^III^‐PBA@Co^II^‐PBA was the best among the samples for driving charge transfer.

**Figure 6:**
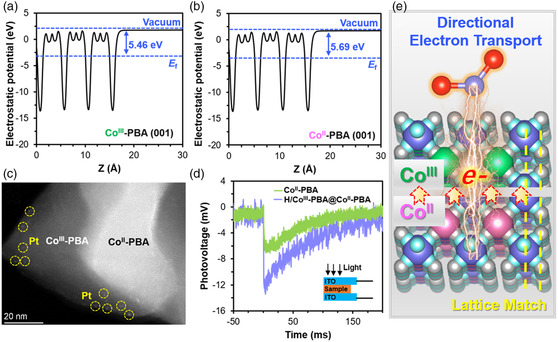
a,b) Calculated electrostatic potentials of the Co^III^‐PBA (001) facet (a) and the Co^II^‐PBA (001) facet (b). c) STEM‐HAADF image of Pt/Co^III^‐PBA@Co^II^‐PBA. d) TPV spectra of Co^II^‐PBA and H/Co^III^‐PBA@Co^II^‐PBA. e) Proposed photocatalytic mechanism of H/Co^III^‐PBA@Co^II^‐PBA with the Ru photosensitizer for visible‐light‐driven reactions.

We also performed theoretical calculations to assess the charge‐transfer pathways in the heterostructures. **Figure** **6**a,b shows that the work function is 5.69 and 5.46 eV for the (001) plane of Co^II^‐PBA and Co^III^‐PBA, respectively. Therefore, upon formation of the Co^III^‐PBA/Co^II^‐PBA heterojunction, electrons redistribute between Co^III^‐PBA and Co^II^‐PBA to establish a built‐in electric field. With this internal electric field, the electrons generated upon light irradiation experience electrostatic attraction and moved from Co^II^‐PBA to Co^III^‐PBA. Both Co^II^‐PBA and Co^III^‐PBA contain active sites that can catalyze the HER and CRR; however, when Co^III^‐PBA is present, it is Co^III^‐PBA, rather than Co^II^‐PBA, which is the ultimate destination of the photogenerated electron before the electron is delivered from the catalyst to the reactant molecule (CO_2_ and H_2_O). Furthermore, the Pt photodeposition experiment and transient photovoltage (TPV) spectra can validate the transfer of photogenerated electrons from Co^II^‐PBA to Co^III^‐PBA. 1) The Pt photodeposition experiment (Figure 6c) shows that the Pt particles prefer to deposit on the Co^III^‐PBA domain because of the accumulation of photogenerated electrons on Co^III^‐PBA to reduce H_2_PtCl_6_ into Pt.^[^ ^33^ ^]^ 2) In the TPV spectra (Figure 6d), both the pure Co^II^‐PBA and H/Co^III^‐PBA@Co^II^‐PBA exhibited a negative signal, indicating that the photogenerated electrons can migrate to the surface under light irradiation.^[^ ^39^ ^]^ About pure Co^II^‐PBA, H/Co^III^‐PBA@Co^II^‐PBA has an increased TPV intensity (Figure 6e and S24, Supporting Information), suggesting the transfer of photogenerated electrons from Co^II^‐PBA to Co^III^‐PBA in the heterostructure.

### 2.5 Significance of Findings

We outline the findings as follows.

#### 2.5.1 Effective Catalyst Design

It is feasible to construct an effective Co^II^‐PBA/Co^III^‐PBA by engineering the oxidation state of the metal sites (i.e., Co in this work) of the two PBAs, wherein the change in the Co valence state helps establish the heterojunction and boosts the charge transfer, thus tuning the geometric and electronic state of the active metal sites to promote the redox reaction. The lattice matching between Co^II^‐PBA and Co^III^‐PBA, for which a similar phase structure and lattice match is critical (Figure 1i), enables the spontaneously ordered alignment to create a robust catalyst with strong interactions between its components, as is evident from the high structural stability and facile electron transfer of the heterostructure.^[^ ^40^ ^]^ In this case, the electronic properties of the heterostructure outweigh the morphology while determining the catalyst activity. The reason includes the performance of the hollow cage‐in‐cage H/Co^III^‐MOF@Co^II^‐MOF is similar to the compact solid S/Co^III^‐MOF@Co^II^‐MOF. Both heterojunctions are similar in size and the thickness of their inner core, and the transport of photogenerated charges thus does not differ substantially from a purely geometrical standpoint. Nevertheless, we anticipate that the cage‐in‐cage MOF@MOF architecture will demonstrate potential applications in other areas that exploit their versatile and highly tunable compositions and structures.^[^ ^41^ ^]^

#### 2.5.2 Delicate MOF@MOF Construction

Epitaxial growth is currently the standard means to create MOF@MOFs, and it is difficult to match the lattice parameters of different MOFs. For transition metals with loosely bonded d electrons (e.g., cobalt in this study), engineering the valence state of the metal makes it possible to construct M^II^‐MOF/M^III^‐MOF heterojunctions. It can be envisioned that when M^II^‐MOF and M^III^‐MOF have similar lattice structures, spontaneous epitaxial growth and regulation of the crystal growth behavior of MOF may occur to create ordered M^II^‐MOF/M^III^‐MOF heterojunctions with intimate contact through a one‐pot procedure. The present findings demonstrate that MOFs can independently adjust their growth behavior to form an intriguing MOF@MOF architecture without additional artificial control over the growth mechanism and kinetics.

## 3 Conclusion

The interface engineering of Co^II^/Co^III^ in heterostructured MOF mesocrystals (Co^III^‐PBA@Co^II^‐PBA) was achieved. The oriented assembly of Co^III^‐PBA nanoparticles occurred around the cubic prisms of Co^II^‐PBA, with Co^III^‐PBA forming a cage structure that enclosed Co^II^‐PBA. DFT calculations and experimental results confirmed that: 1) Co^III^‐PBA has a stronger affinity for CO_2_ than Co^II^‐PBA and 2) the photogenerated electrons can be quickly transferred from Co^II^‐PBA to Co^III^‐PBA through the built‐in electric field in the heterojunction of Co^III^‐PBA@Co^II^‐PBA. In the photocatalytic CO_2_‐to‐syngas process, the electronics of the Co site in Co^III^‐PBA@Co^II^‐PBA seem to be more critical than morphology, as S/Co^III^‐PBA@Co^II^‐PBA and H/Co^III^‐PBA@Co^II^‐PBA have similar photocatalytic performances, while both strongly outperform many recently reported photocatalysts for solar‐driven syngas production. The excellent syngas production can be attributed to the directional transfer of high‐energy electrons to the more reactive metal centers in the Co^III^‐PBA.

## 4 Experimental Section

4.1

4.1.1

##### Materials

Chemicals in experiments were of analytical grade, with potassium hexacyanoferrate(III) (K_3_Fe(CN)_6_), cobalt(II) acetate tetrahydrate (C_4_H_6_CoO_4_·4H_2_O), trisodium citrate dihydrate (Na_3_C_6_H_5_O_7_·2H_2_O), and acetonitrile (CH_3_CN) purchased from Sinopharm Chemical Reagent Co. Ltd., and triethanolamine (C_6_H_15_NO_3_) and ethanol (C_2_H_5_OH) tris(bipyridine)ruthenium(II) chloride ([Ru(bpy)_3_]Cl_2_·6H_2_O) from Aladdin Reagent Co. Ltd. Deionized (DI) water was used in all experiments.

##### Synthesis of Co‐PBA

Solution A is prepared by dissolving cobalt(II) acetate tetrahydrate and trisodium citrate dihydrate (0.3 g) in DI water (40 mL). Potassium hexacyanoferrate(III) (0.2 g) was dissolved in DI water (60 mL) to form solution B. Dropwise addition of solution B to solution A was accomplished under magnetic stirring, and the mixture was stirred for another 24 h at ambient temperature. The precipitate was collected by centrifugation, washed with DI water and ethanol, and dried under a vacuum. The obtained product depended on the dosage of cobalt(II) acetate tetrahydrate in the starting materials, about 0.03, 0.23, and 0.33 g for Co^II^‐PBA, S/Co^III^‐PBA@Co^II^‐PBA, and H/Co^III^‐PBA@Co^II^‐PBA, respectively.

## Conflict of Interest

The authors declare no conflict of interest.

## Supporting information

Supplementary Material

## Data Availability

The data that support the findings of this study are available from the corresponding author upon reasonable request.
