# Supplementary material for: Ordered CoIII‐MOF@CoII‐MOF Heterojunction for Highly Efficient Photocatalytic Syngas Production
Source: Small Sci. 2023 Feb 28;3(4):2200085. doi: 10.1002/smsc.202200085 (PMC11935870; doi:10.1002/smsc.202200085)
Supplement: Supplementary file 1 — Supplementary Material [file SMSC-3-2200085-s001.pdf]

Supporting Information for

Ordered Co^{III}-MOF@Co^{II}-MOF Heterojunction for Highly Efficient Photocatalytic Syngas Production

Mingxiong Lin,+[a] [b] Weishan Jiang,+[a] [b] Tingshi Zhang,[a] [b] Bixia, Yang,[a] [b] Zanyong Zhuang,[a] [b] Yan Yu*[a] [b]*

(+: These authors contributed equally to this work)

[a]*College of Materials Science and Engineering, Fuzhou University, New Campus, Minhou, Fujian Province 350108, China*
[b]*Key Laboratory of Advanced Materials Technologies, Fuzhou University, Fuzhou 350108, China*

***Corresponding Authors**

E-mail: zyzhuang@fzu.edu.cn, yuyan@fzu.edu.cn

Materials characterization

X-ray diffraction patterns were recorded to determine the phase of catalysts on X-ray diffractometer (XRD, PANalytical X'pert MPD). With scanning electron microscope (SEM, Philips XL30) and transmission electron microscope (TEM, FEI Tecnai G2 F20) equipped with dispersive spectroscopy (EDS), the size, morphology and chemical components of samples were investigated. X-ray photoelectron spectra (XPS) and Fourier-transform infrared (FT-IR) spectra were recorded on PHI 5000 VersaProbe spectrometer, Nicolet 5700 spectrometer, respectively. In addition, UV-Vis diffuse reflectance spectra (DRS) were recorded using $BaSO_4$ as the reference, on Varian Cary 500 UV-Vis-NIR spectrometer equipped with an integrating sphere. Micromeritics ASAP 2020 apparatus was applied to investigate the Brunauer–Emmett–Teller (BET) surface area of catalysts. Photoluminescence (PL) spectra and time-resolved PL (TRPL) spectra were collected on Hitachi F-7000 fluorescence spectrophotometer at 410 nm excitation, and on Edinburgh FLS-920 spectrofluorometer, respectively. Transient photocurrent measurements were collected on CHI-660C electrochemical workstation (Chenhua, China). Electrochemical impedance spectroscopy (EIS) and Mott–Schottky measurements were run on Autolab PGSTAT204 electrochemical workstation. The gas production was analyzed on Agilent 7890B gas chromatograph (GC) equipped with thermal conductivity detector (TCD) and TDX-01 column using Ar as the carrier gas. ^1H nuclear magnetic resonance (^1H-NMR) was recorded on Bruker DPX 400 spectrometer. The transient photovoltage (TPV) spectra was run on CEL-SPS1000 instrument. Bruker INVENIO R FT-IR spectrometer equipped with an in situ diffuse reflectance cell (Harrick) helps to record the *in-situ* FT-IR spectra.

Photocatalytic CRR and HER

The photocatalytic activities of catalysts were evaluated in a quartz reactor, which contains 0.5 mg catalyst and 8 mg $[Ru(bpy)_3]Cl_2·6H_2O$ in 6 mL mixed solvent with 3:2:1 (v/v) CH_3CN/H_2O/TEOA. The reactor was evacuated and purged with CO_2 for three cycles, and then purged again by high-purity CO_2 for 30 min. The reactions were run under light irradiation using a 300 W Xenon lamp with a 420 nm cutoff filter under stirring. The temperature of catalytic system was kept at 301–303 K using circulating cooling water and heater, with the gaseous products to be analyzed by gas chromatography (Agilent 7890B).

Photoelectrochemical Activity Test

Photoelectrochemical tests were run in a standard three-electrode quartz cell, with FTO glass (with the photocatalyst coated on a 0.25 cm^2 area), Pt, and Ag/AgCl as The working, counter, and reference

electrodes, respectively. The Mott–Schottky tests used a Na_2SO_4 solution (0.1 M), the EIS tests used a mixed aqueous solution of 5 mM $K_3[Fe(CN)_6]$/5 mM $K_4[Fe(CN)_6]$/0.1 M KCl, and they were both run on a ParSTAT MC electrochemical workstation (Princeton Applied Research, Co.) Transient photocurrent response was recorded under irradiation with a Xenon lamp, in a mixed solution containing 20 mg $[Ru(bpy)_3]Cl_2{\cdot}6H_2O$, 10 mL TEOA, 20 mL H_2O, and 30 mL CH_3CN on a CHI-660E electrochemical analyzer.

DFT Computation Details

Density functional theory (DFT) calculations were run using Vienna Ab Initio Package, while ionic cores and consider valence electrons were described by the projected augmented wave potentials with kinetic energy cutoff of 450 eV. The methods to check the partial occupancies of the Kohn−Sham orbitals, the electronic energy and the Geometry optimization follows other reports previously.[1] A surface structure of 2×2×1 Monkhorst pack K-point sampling is set for the Brillouin zone integral. The calculation of adsorption energies (E_{ads}) was via equation of $E_{ads}=E_{ad/sub}-E_{ad}-E_{sub}$ following our previous reports.[2] Likewise, the evaluation of work function was based on equation of $\Phi= E_{vac}-E_F$. [3]

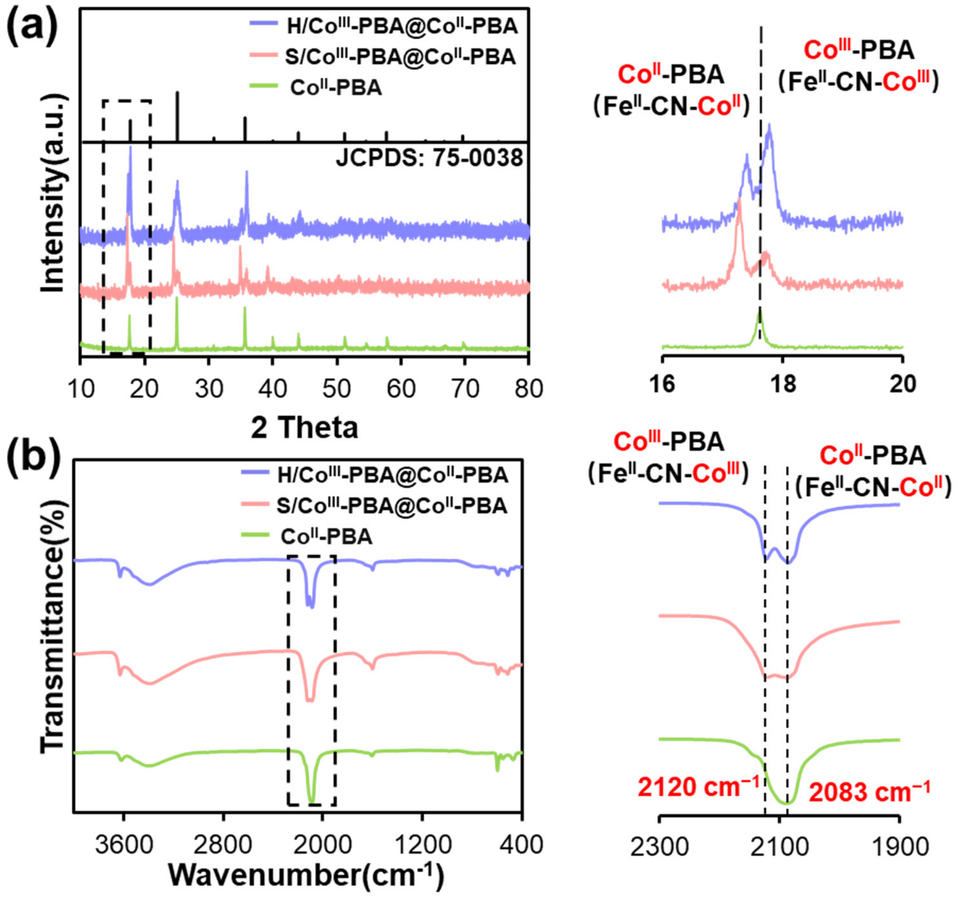

Figure S1. (a) XRD patterns and (b)FI-IR spectra of Co^{II}-PBA, S/Co^{III}-PBA@Co^{II}-PBA and H/Co^{III}-PBA@Co^{II}-PBA, respectively.

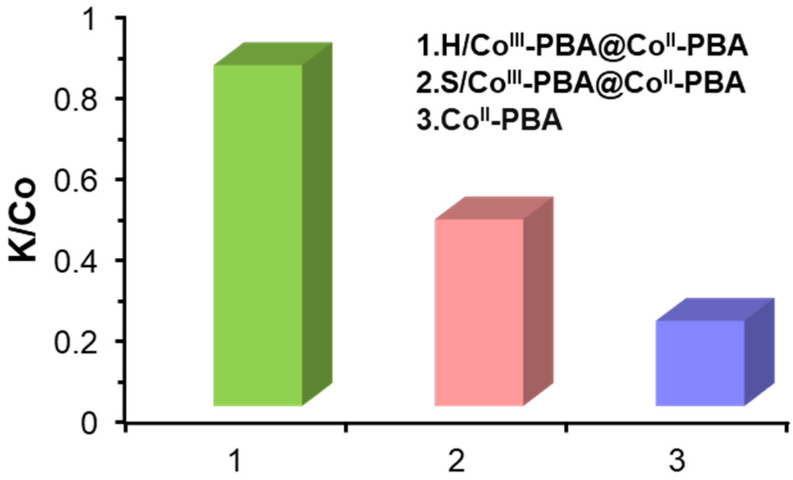

Figure S2. Elemental analysis and EDS spectrometry showing reduced K content compared to the pristine Co^{II}-PBA ($K_2Co[Fe(CN)_6]$).

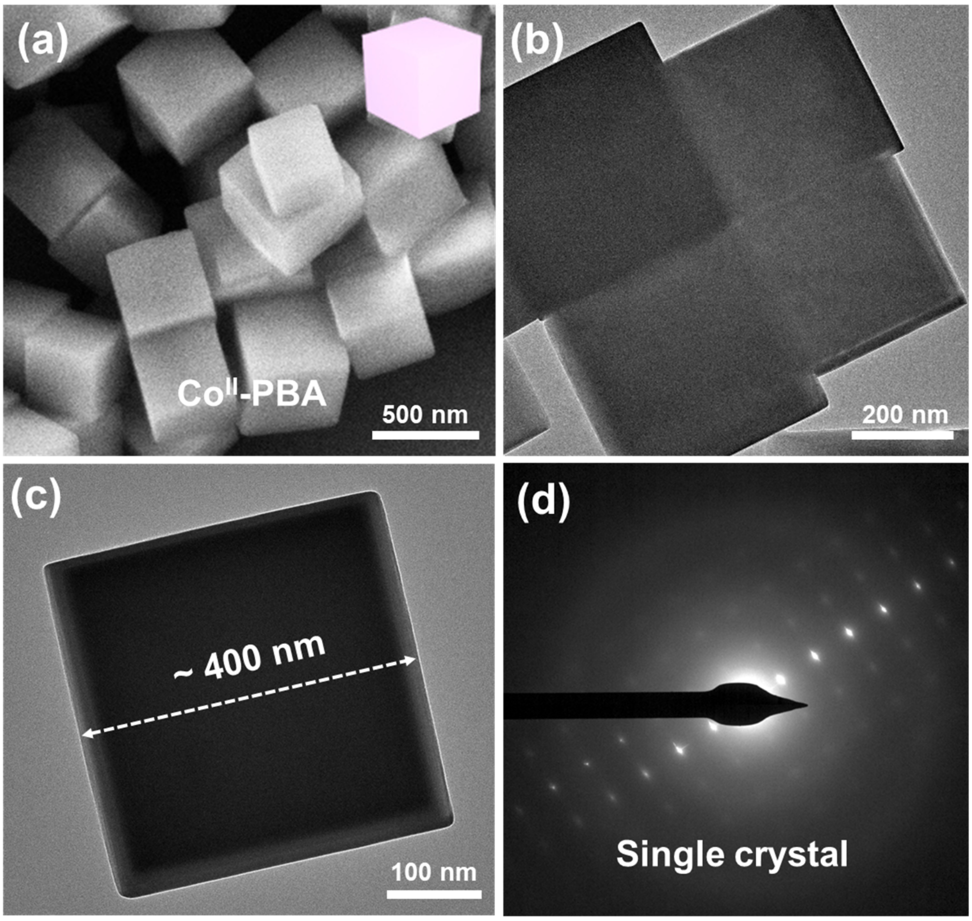

Figure S3. (a) SEM image, (b, c) TEM images and (d) SEAD pattern of Co^{II}-PBA.

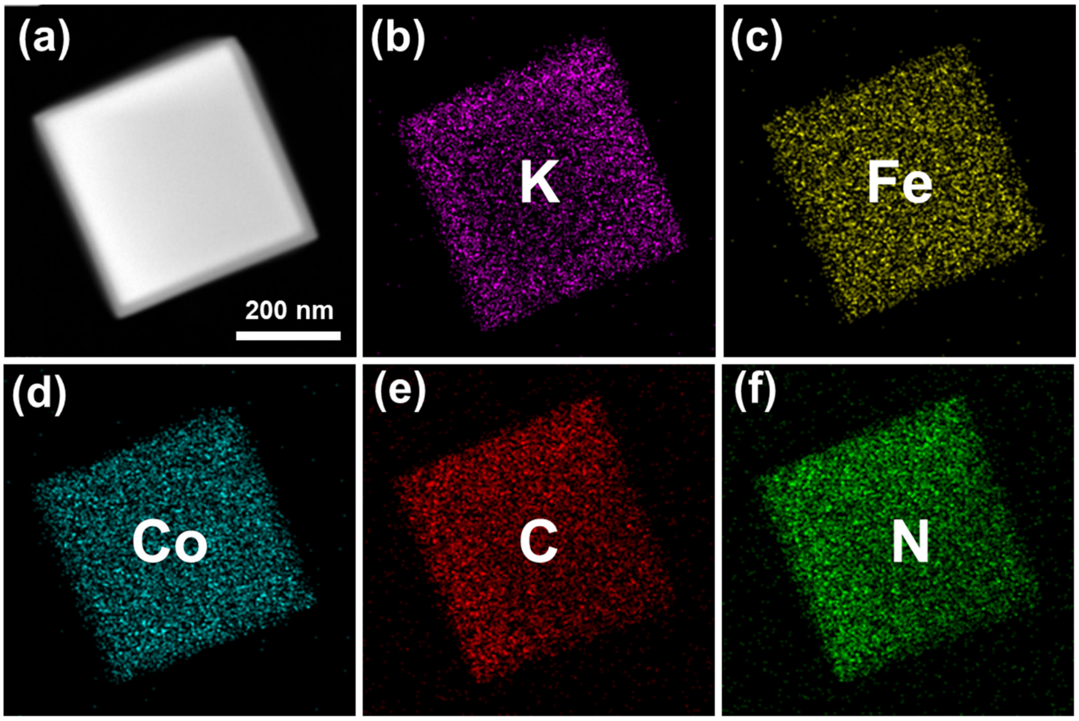

Figure S4. (a) HAADF-STEM image and (b-f) element mappings of Co^{II}-PBA.

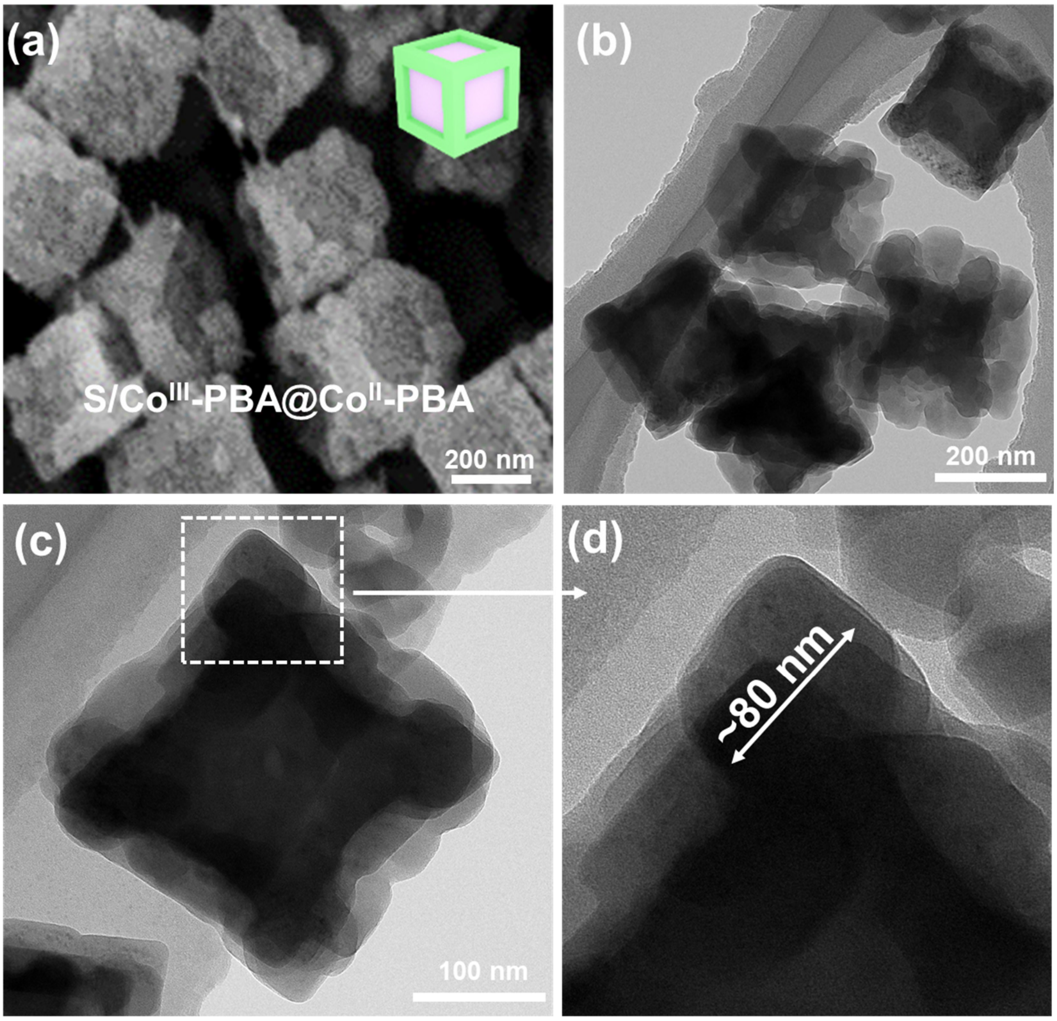

Figure S5. (a) SEM image and (b-d) TEM of images of S/Co^{III}-PBA@Co^{II}-PBA.

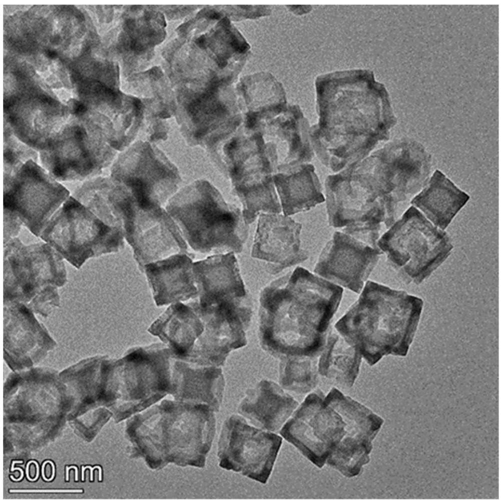

Figure S6. TEM image of H/Co^{III}-PBA@Co^{II}-PBA.

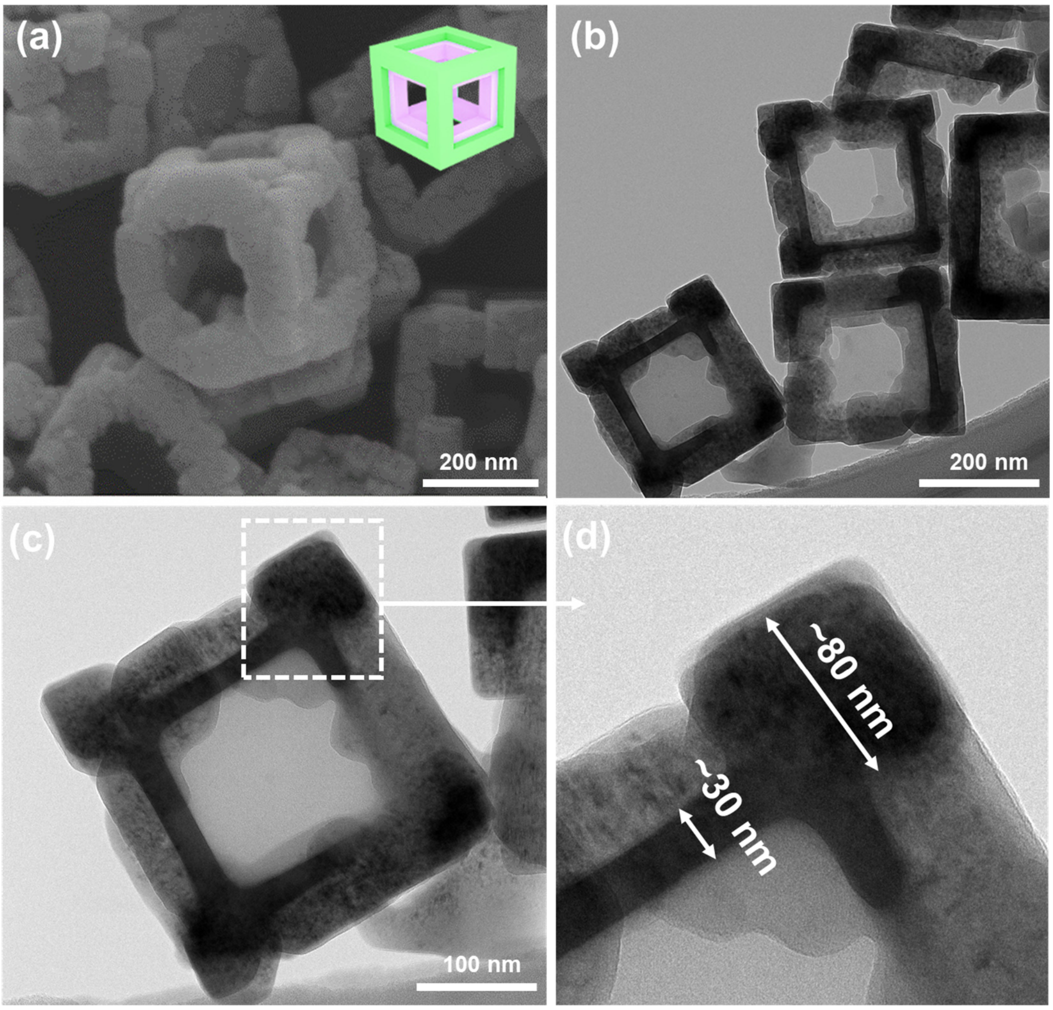

Figure S7. (a) SEM image and (b-d) TEM of images of H/Co^{III}-PBA@Co^{II}-PBA.

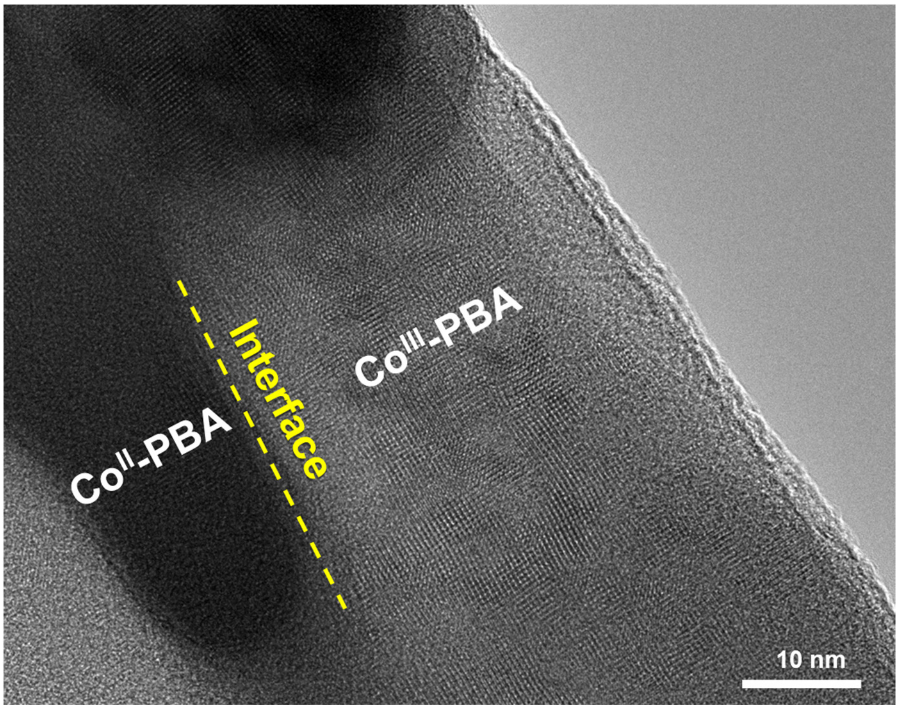

Figure S8. HRTEM of image of H/Co^{III}-PBA@Co^{II}-PBA.

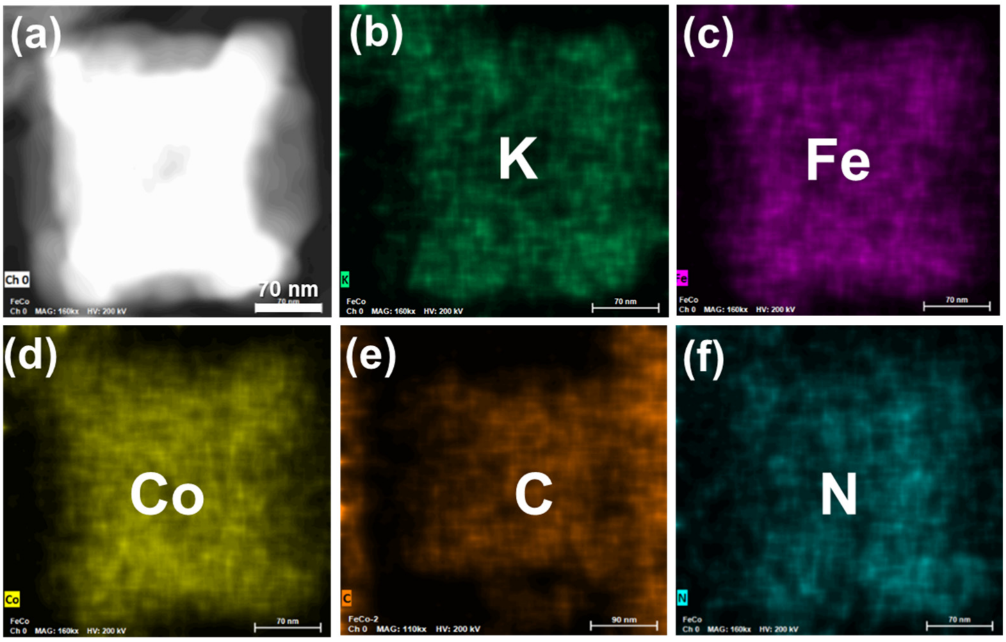

Figure S9. (a) HAADF-STEM image and (b-f) element mappings of S/Co^{III}-PBA@Co^{II}-PBA.

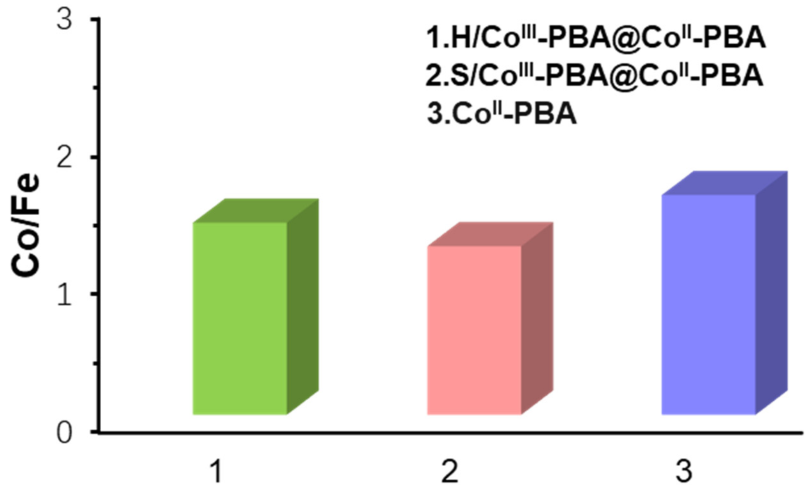

Figure S10. EDS spectrometry of Co^{II}-PBA, S/Co^{III}-PBA@Co^{II}-PBA and H/Co^{III}-PBA@Co^{II}-PBA.

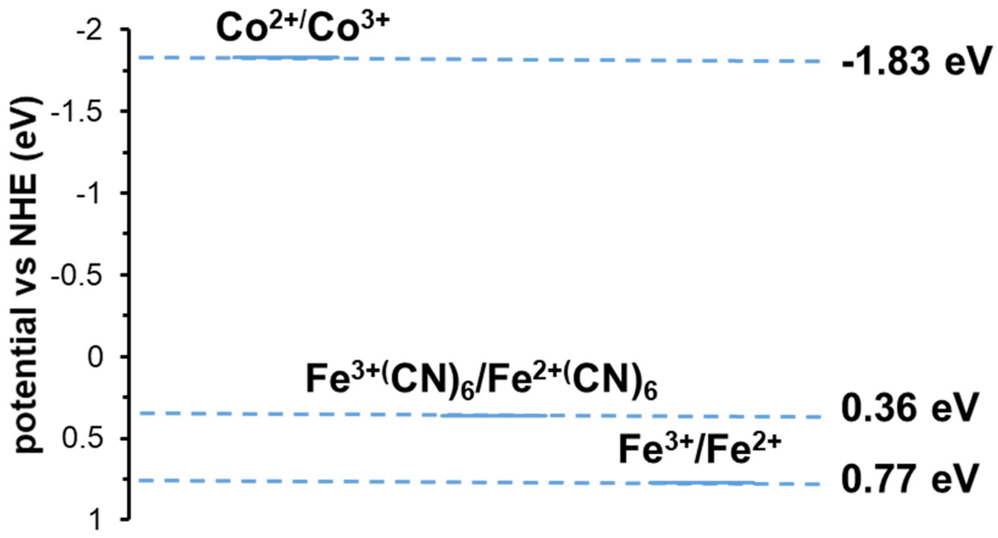

Figure S11. The redox potential of Co^{2+}/Co^{3+}, $[Fe^{3+}(CN)_6]^{3-}/[Fe^{2+}(CN)_6]^{4-}$ and Fe^{3+}/Fe^{2+}, respectively.

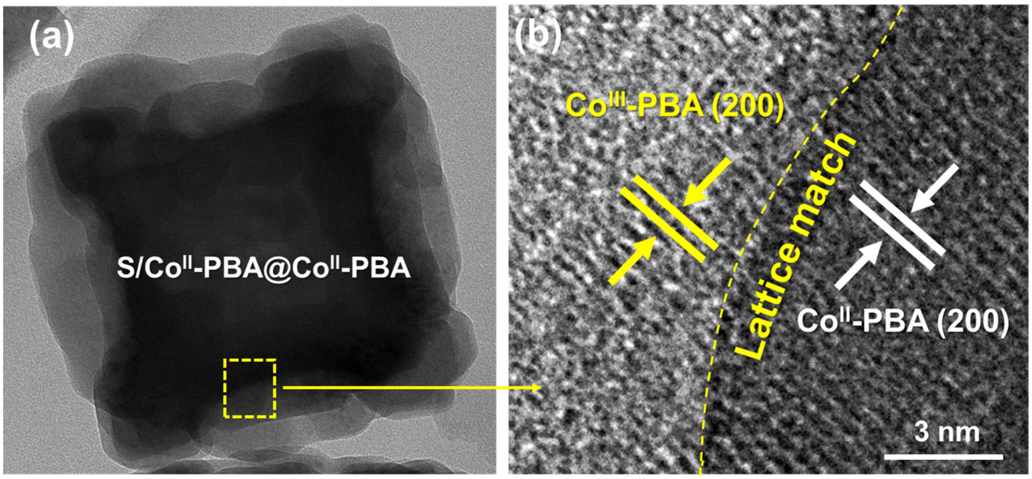

Figure S12. (a) TEM and (b) HRTEM images of S/Co^{III}-PBA@Co^{II}-PBA.

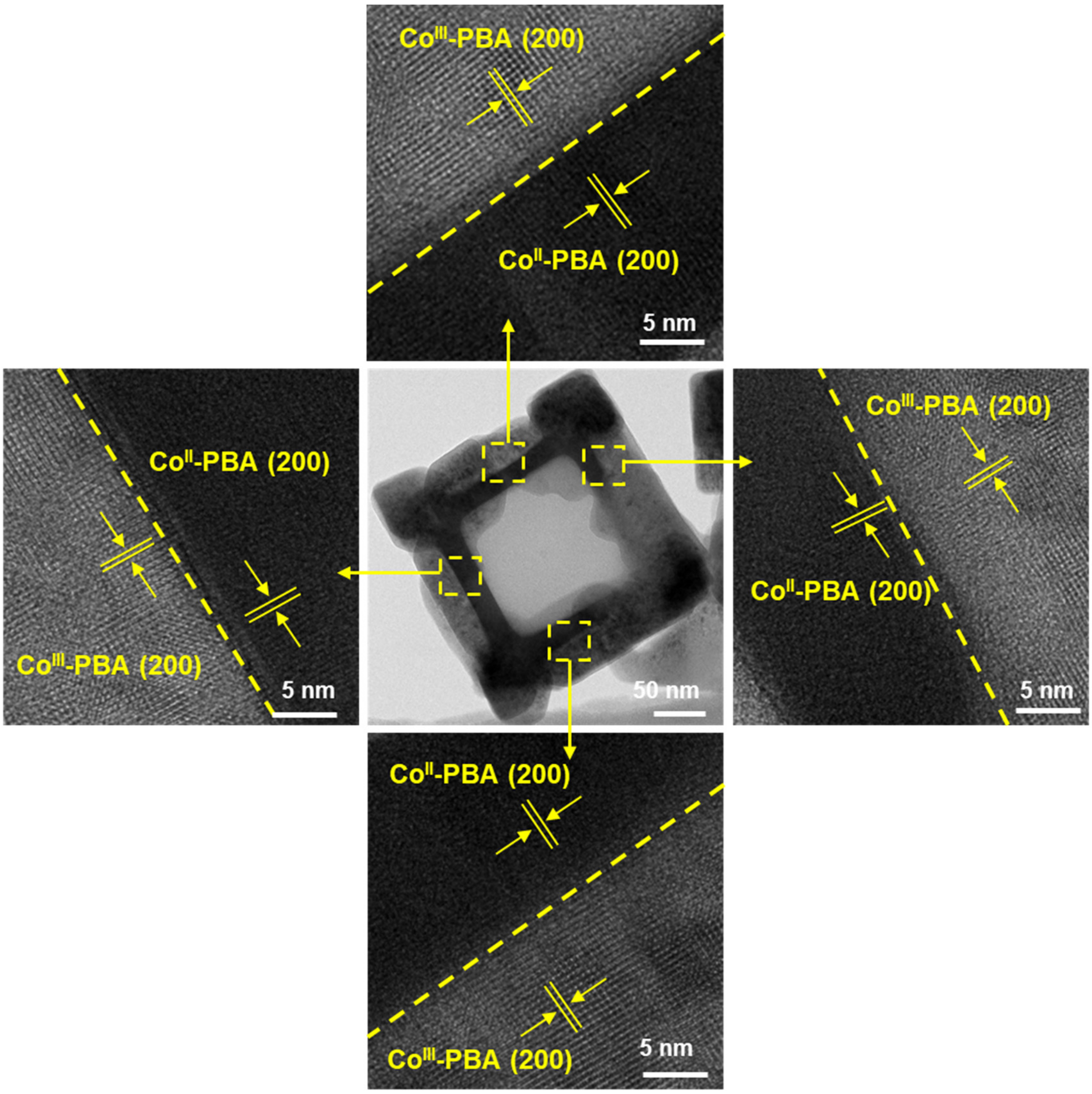

Figure S13. TEM and HRTEM images of H/Co^{III}-PBA@Co^{II}-PBA.

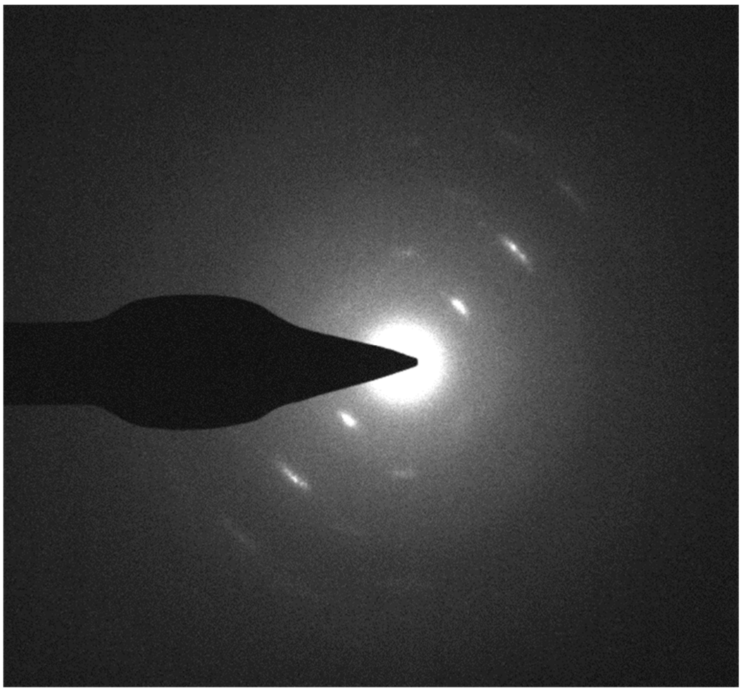

Figure S14. SEAD pattern of S/Co^{III}-PBA@Co^{II}-PBA.

Table S1. BET surface areas and CO_2-uptake capability of the catalysts
.

Catalysts	BET surface areas (m^2 g^{-1})	CO_2-uptake capability (cm^3 g^{-1})
Co^{II}-PBA	1.4	2.7
S/Co^{III}-PBA@Co^{II}-PBA	156.6	27.6
H/Co^{III}-PBA@Co^{II}-PBA	162.4	29.1

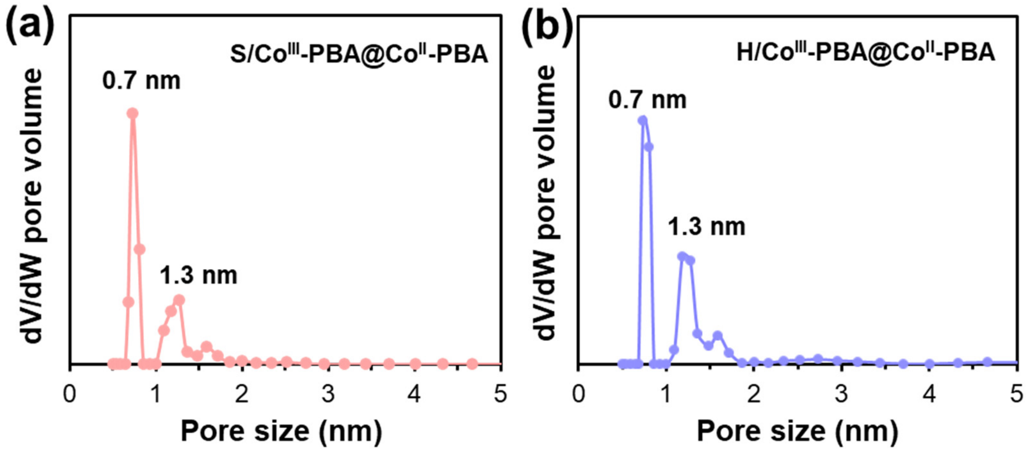

Figure S15. Pore size distribution diagram of (a) S/Co^{III}-PBA@Co^{II}-PBA and (b) H/Co^{III}-PBA@Co^{II}-PBA.

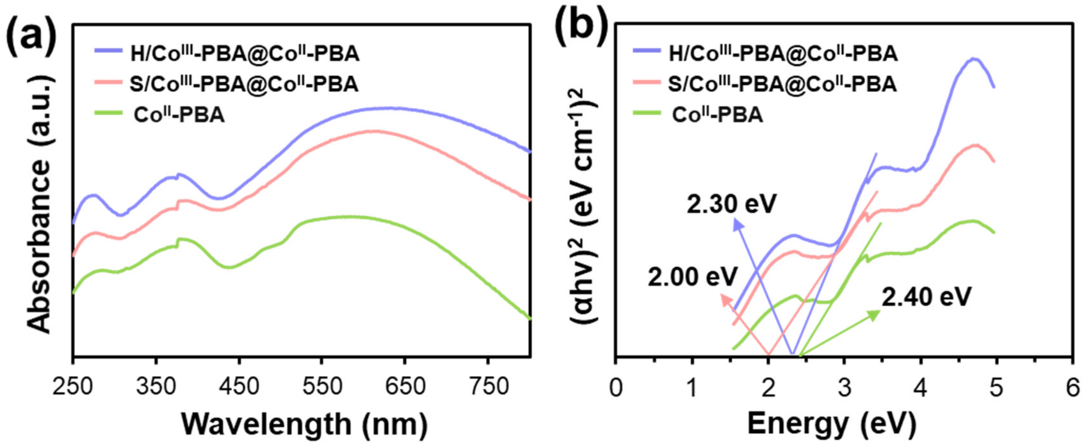

Figure S16. (a) UV–Vis spectroscopy and (b) Tauc plots of Co^{II}-PBA, S/Co^{III}-PBA@Co^{II}-PBA and H/Co^{III}-PBA@Co^{II}-PBA.

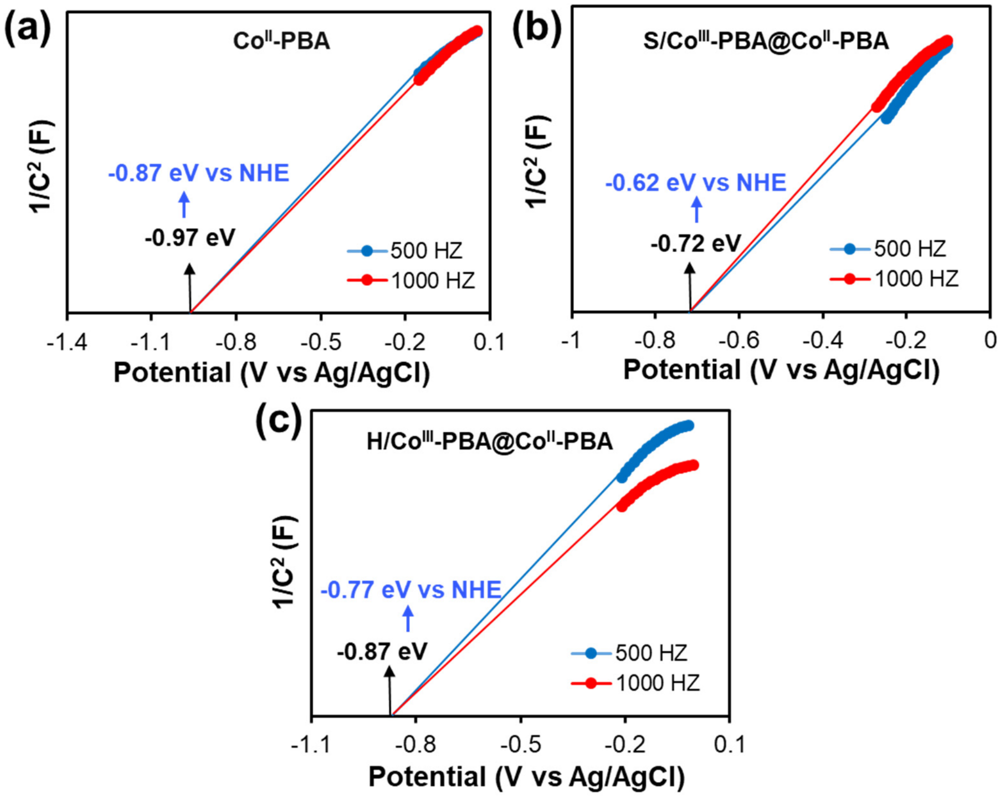

Figure S17. Mott–Schottky plots of (a) Co^{II}-PBA, (b) S/Co^{III}-PBA@Co^{II}-PBA and (c) H/Co^{III}-PBA@Co^{II}-PBA.

Table S2. CB and VB positions (V, vs. NHE, pH=7) of the PBA photocatalysts.

Catalysts	Co^{II}-PBA	S/Co^{III}-PBA@Co^{II}-PBA	H/Co^{III}-PBA@Co^{II}-PBA
Eg (eV)	2.40	2.00	2.30
CB (V)	-0.87	-0.62	-0.77
VB (V)	+1.53	+1.38	+1.53

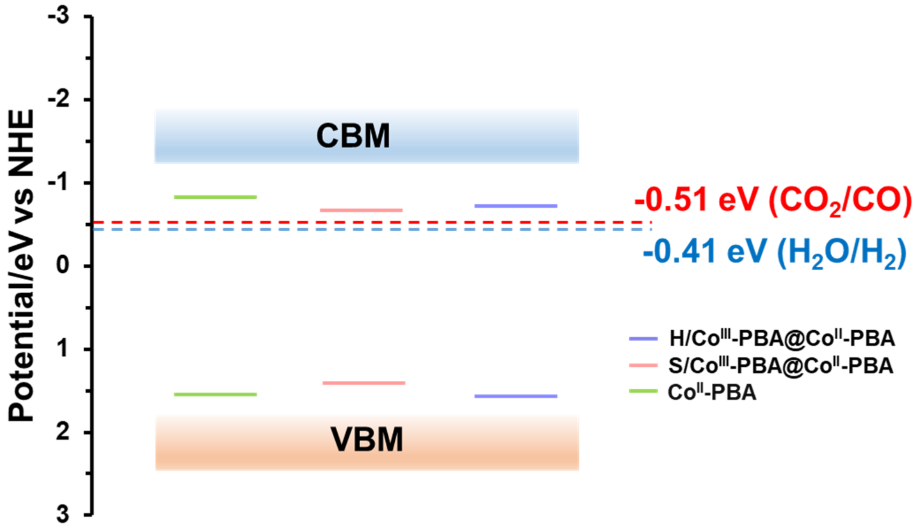

Figure S18. Energy band diagram of the photocatalyst, respectively.

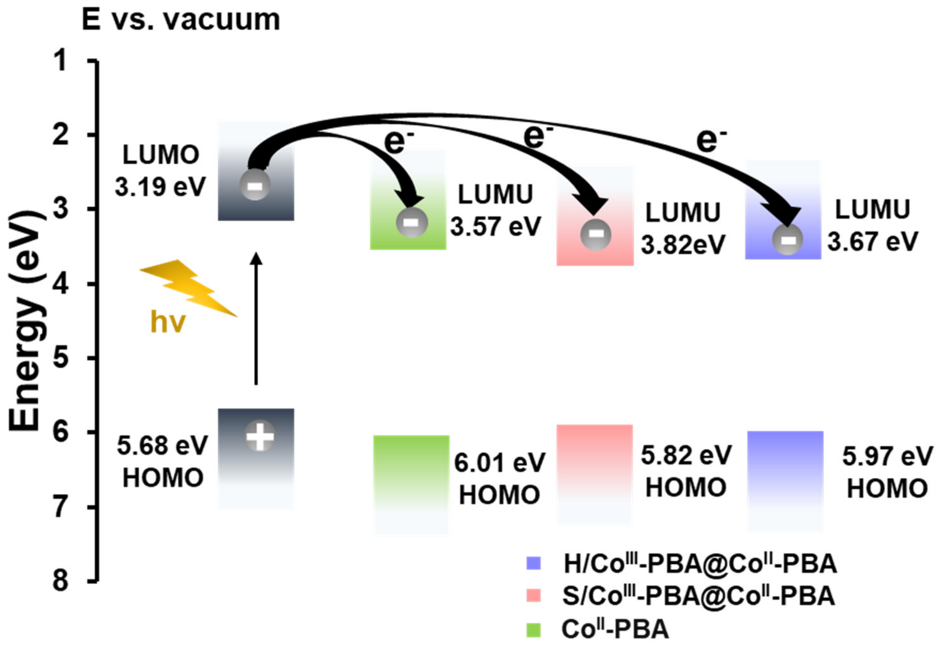

Figure S19. Energy band diagram of the photocatalyst, respectively. As the CBM relative to vacuum level (E_{LUMO}) is calculated to be 3.57, 3.82 and 3.67 eV according to E_{LUMO}= E_{CBM} + 4.44 eV, respectively,[4] the valence band minimum relative to vacuum level (E_{HOMO}) of Co^{II} PBA, S/Co^{III} PBA@Co^{II} PBA and H/Co^{III} PBA@Co^{II} PBA can be worked out to be 6.01, 5.82 and 5.97 eV, respectively.

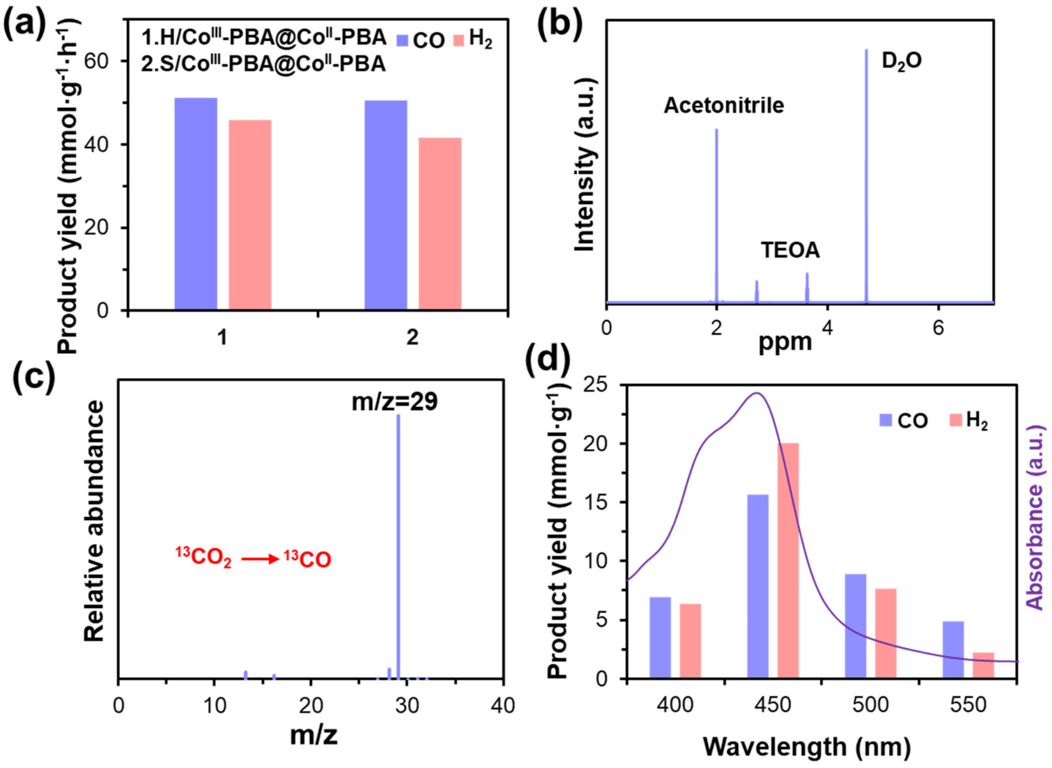

Figure S20. (a) Photocatalytic CO_2-to-syngas performance over S/Co^{III}-PBA@Co^{II}-PBA and H/Co^{III}-PBA@Co^{II}-PBA; (b) ^{1}H NMR spectrum of the liquid phase from the reaction system after visible-light irradiation for 3 h; (c) Mass spectrum of ^{13}CO (m/z = 29) produced over H/Co^{III}-PBA@Co^{II}-PBA in the photocatalytic reduction of $^{13}CO_2$; (d) Wavelength dependence of production of CO and H_2 in 2 h reaction.

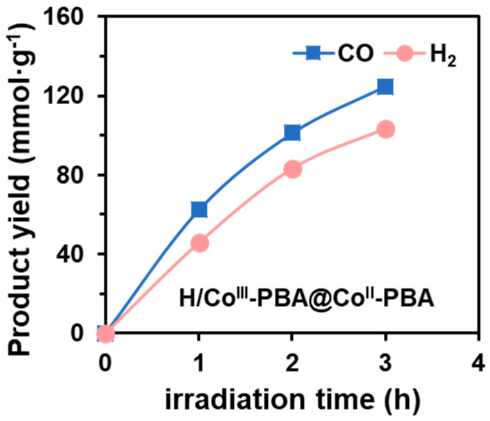

Figure S21. The time-dependent syngas production of H/Co^{III}-PBA@Co^{II}-PBA.

Table S3. CRR performance of H/Co^{III}-PBA@Co^{II}-PBA and other reported catalysts[a]

Catalysts	Photosensitizer sacrificial agent	CO yield (mmol $g^{-1}h^{-1}$)	H_2 yield (mmol $g^{-1}h^{-1}$)	Irradiation condition	Reference
Co_6-MOF	$Ru(bpy)_3^{2+}$ TEOA	4.37	3.13	λ ≥ 420 nm (Xe lamp)	[5]
Co-ZIF-9	$Ru(bpy)_3^{2+}$ TEOA	8.36	5.98	λ ≥ 420 nm (Xe lamp)	[6]
ZIF-67	$Ru(bpy)_3^{2+}$ TEOA	29.6	10.29	λ ≥ 420 nm (Xe lamp)	[7]
ZIF-67-3	$Ru(bpy)_3^{2+}$ TEOA	3.89	2.33	λ≥420 nm (5W LED)	[8]
C-$BMZIF_{3:1}$	$Ru(bpy)_3^{2+}$ TEOA	11.4	7.6	λ ≥ 420 nm (Xe lamp)	[9]
Co_3O_4 HNS	$Ru(bpy)_3^{2+}$ TEOA	1.99	1.53	λ≥400 nm (5W LED)	[10]
ZIF-67@α-TiO_2	$Ru(bpy)_3^{2+}$ TEOA	10.95	5.35	λ ≥ 420 nm (Xe lamp)	[11]
Co_3O_4-NS	$Ru(bpy)_3^{2+}$ TEOA	23.00	16.12	λ ≥ 400 nm (Xe lamp)	[12]
Fe-SAs/N-C	[Ru $Ru(bpy)_3^{2+}$ TEOA	4.5	4.95	λ ≥ 420 nm (Xe lamp)	[13]
POP_n-Fe	[Ru $Ru(bpy)_3^{2+}$ TEOA	3.04	3.75	λ ≥ 420 nm (Xe lamp)	[14]
CoO	[Ru $Ru(bpy)_3^{2+}$ TEOA	11.56	4.17	λ ≥ 420 nm (Xe lamp)	[15]
Cu_2S@ROH-$NiCo_2O_3$	[Ru $Ru(bpy)_3^{2+}$ TEOA	7.1	2.8	λ ≥ 400 nm (Xe lamp)	[16]
Co@COF-TVBT-Bpy	[Ru $Ru(bpy)_3^{2+}$ TEOA	1.13	1.16	λ ≥ 420 nm (LED lamp)	[17]
H/Co^{III}-PBA@Co^{II}-PBA	$Ru(bpy)_3^{2+}$ TEOA	50.56	41.63	λ ≥ 420 nm (Xe lamp)	[This work]

[a]All reactions were carried out in CH_3CN/H_2O

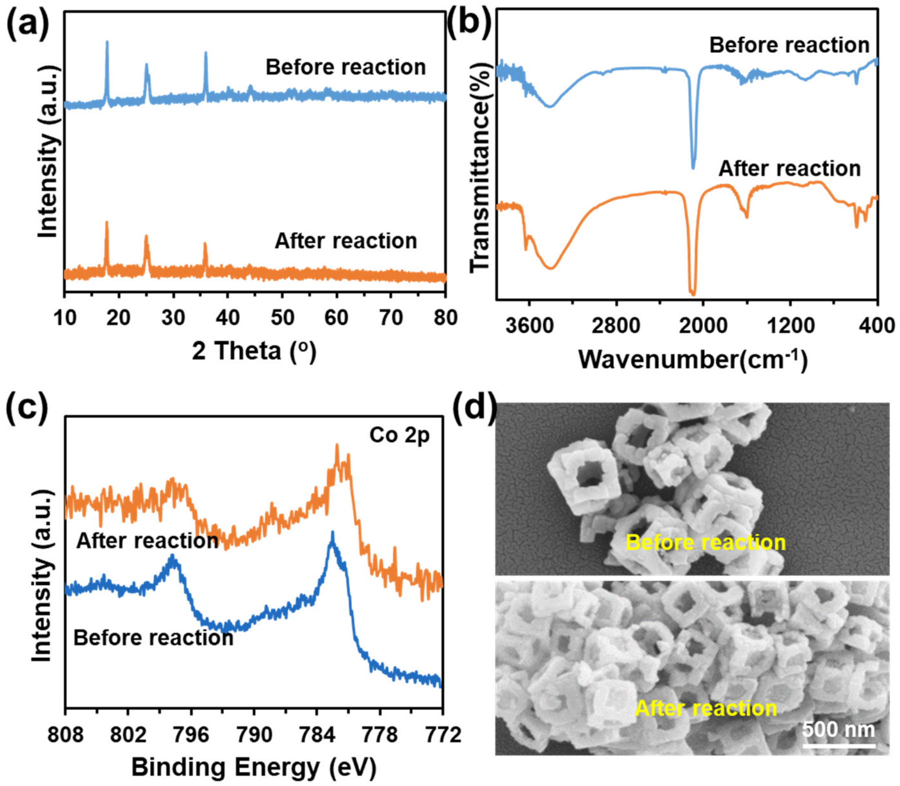

Figure S22. (a) XRD pattern, (b) FI-IR spectra, (c) XPS spectra of Co 2p and (d) SEM images of H/Co^{III}-PBA@Co^{II}-PBA before and after reaction.

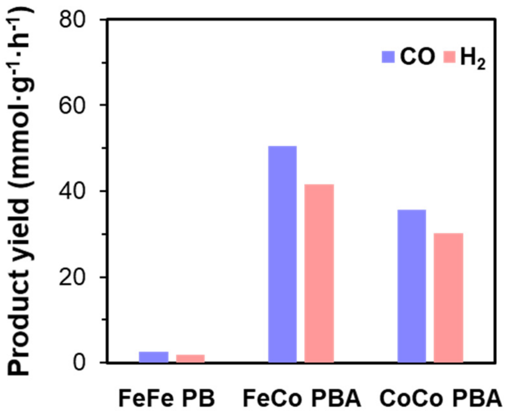

Figure S23. CO_2 photoreduction performance over FeFe PB (Without Co), FeCo PBA (H/Co^{III}-PBA@Co^{II}-PBA) and CoCo PBA (Without Fe), respectively.

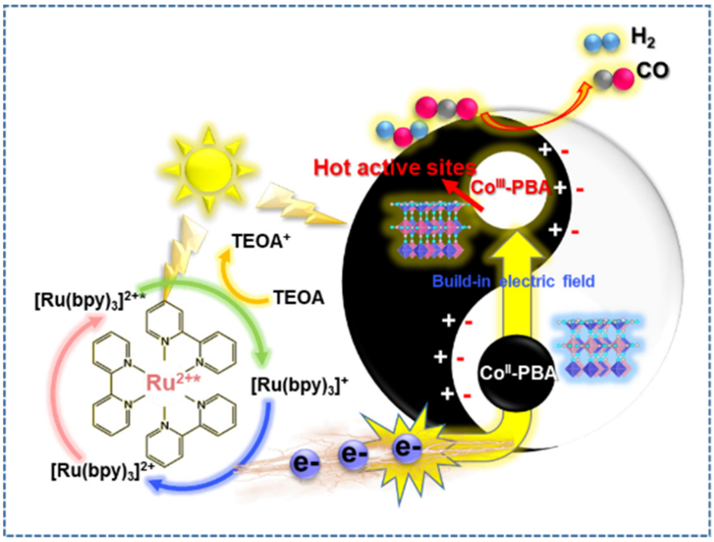

Figure S24. Proposed photocatalytic mechanism of H/Co^{III}-PBA@Co^{II}-PBA with the Ru photosensitizer for visible-light driven.

.

References

[1] Y. Zhu, S. Qiu, W. Tang, F. Deng, F. Ma, Y. Zheng, H. Xie, *Chem. Eng. J.* **2022**, *437*, 135480.
[2] M. Lin, Y. Luo, T. Zhang, X. Shen, Z. Zhuang, Y. Yu, *ACS Appl. Mater. Interfaces* **2022**, *14*, 52868.
[3] M. Lin, R. Cao, Y. Luo, T. Zhang, Z. Zhuang, Y. Yu, *ACS Appl. Energy Mater.* **2022**, *5*, 2161.
[4] L. Chen, X. Wang, Y. Chen, Z. Zhuang, F.-F. Chen, Y.-J. Zhu, Y. Yu, *Chem. Eng. J.* **2020**, *402*, 125922.
[5] J. Zhao, Q. Wang, C. Sun, T. Zheng, L. Yan, M. Li, K. Shao, X. Wang, Z. Su, *J. Mater. Chem. A* **2017**, *5*, 12498.
[6] S. Wang, W. Yao, J. Lin, Z. Ding, X. Wang, *Angew. Chem.* **2014**, *126*, 1052.
[7] J. Qin, S. Wang, X. Wang, *Appl. Catal. B* **2017**, *209*, 476.
[8] M. Wang, J. Liu, C. Guo, X. Gao, C. Gong, Y. Wang, B. Liu, X. Li, G. G. Gurzadyan, L. Sun, *J. Mater. Chem. A* **2018**, *6*, 4768.
[9] Q. Mu, W. Zhu, G. Yan, Y. Lian, Y. Yao, Q. Li, Y. Tian, P. Zhang, Z. Deng, Y. Peng, *J. Mater. Chem. A* **2018**, *6*, 21110.
[10] J.-T. Ren, Y.-L. Zheng, K. Yuan, L. Zhou, K. Wu, Y.-W. Zhang, *Nanoscale* **2020**, *12*, 755.
[11] H. Wang, D. Wu, C. Yang, H. Lu, Z. Gao, F. Xu, K. Jiang, *Journal of CO_2 Utilization* **2019**, *34*, 411.
[12] W. Chen, B. Han, C. Tian, X. Liu, S. Liang, H. Deng, Z. Lin, *Appl. Catal. B* **2019**, *244*, 996.
[13] Z. Wang, J. Yang, J. Cao, W. Chen, G. Wang, F. Liao, X. Zhou, F. Zhou, R. Li, Z.-Q. Yu, *ACS nano* **2020**, *14*, 6164.
[14] X. Yao, K. Chen, L.-Q. Qiu, Z.-W. Yang, L.-N. He, *Chem. Mater.* **2021**, *33*, 8863.
[15] H. Yang, D. Yang, X. Wang, *Angew. Chem. Int. Ed.* **2020**, *59*, 15527.
[16] L. Li, X. Dai, D. L. Chen, Y. Zeng, Y. Hu, X. W. Lou, *Angew. Chem. Int. Ed.* **2022**, *61*, e202205839.
[17] J.-X. Cui, Y.-M. Fu, B. Meng, J. Zhou, Z.-Y. Zhou, S.-M. Liu, Z.-M. Su, *J. Mater. Chem. A* **2022**. DOI: https://doi.org/10.1039/D2TA02648A
